# The Influence of Humic Substances and Auxin-Producing Bacteria on *Acer saccharinum* Plants in Relation to Auxin-Humate Binding

**DOI:** 10.3390/ijms27125494

**Published:** 2026-06-18

**Authors:** Maxim Timergalin, Ruslan Ivanov, Gleb Zaitsev, Nadezhda Ryazanova, Rimma Abdullina, Sergey Chetverikov, Zinnur Shigapov, Leila Timergalina, Aleksey Nazarov, Edward Khamitov, Valeria Kayukova, Sergey Khursan, Guzel Kudoyarova

**Affiliations:** 1 Ufa Institute of Biology, Ufa Federal Research Centre, Russian Academy of Sciences, Prospekt Oktyabrya 69, 450054 Ufa, Russia; timermax@mail.ru (M.T.); ivanovirs@mail.ru (R.I.); che-kov@mail.ru (S.C.); leinaz@mail.ru (L.T.); guzel@anrb.ru (G.K.); 2 South Ural Botanical Garden-Institute, Ufa Federal Research Center, Russian Academy of Sciences, 450080 Ufa, Russia; ryazanova@mail.ru (N.R.); rimmaabdullina@yandex.ru (R.A.); shigapov@anrb.ru (Z.S.); 3 Department of Environment and Rational Use of Natural Resources, Faculty of Business Ecosystem and Creative Technologies, Ufa State Petroleum Technological University, ul. Kosmonavtov 1, 450064 Ufa, Russia; nazarovam1501@gmail.com; 4 Ufa Institute of Chemistry, Ufa Federal Research Centre, Russian Academy of Sciences, Prospekt Oktyabrya 69, 450054 Ufa, Russia; khamitovem@gmail.com (E.K.); khursansl@gmail.com (S.K.); 5 LLC Bioprominvest, Zavodskaya Str., 20, 450097 Ufa, Russia; kayukovavg@gmail.com

**Keywords:** silver maple (*Acer saccharinum* L.), humic substances, auxin, indole-3-acetic acid, abscisic acid, cytokinin, root branching, plant-growth promoting bacteria, molecular modelling, optical absorption spectrometry

## Abstract

Silver maple is a fast-growing, adaptable tree that often frequents wet places and thus can play an important ecological role in replanting schemes. For this, robust, high-quality seedlings are essential. In other tree species, improved seedling quality has been achieved by treating with a combination of humic substances (HSs) and bacterial strains capable of synthesizing auxin phytohormone; the benefit being attributed, without clear supporting evidence, to changes in phytohormone concentrations in the plant. To clarify the uncertainty, we conducted assays of hormones in silver maple seedlings treated with HSs and appropriate bacteria. We hypothesized that any positive additive effects between HSs and bacteria may be due to the ability of HSs to bind phytohormones. This hypothesis was tested and confirmed by using optical absorption spectra of auxins, humic acids, and their combination, as well as by modeling their interactions. The combination of humic substances and bacteria resulted in an approximately 1.5-fold increase in auxin content in roots, accompanied by a marked increase in root weight and length. We suggest this is likely the outcome of HSs binding to bacterial auxins and delivering them to plant roots. Concentrations of cytokinins and abscisic acid also changed under these treatments, which may help explain observed increases in photosynthesis and improved water balance.

## 1. Introduction

The diversity of maple species explains their presence in a wide range of contrasting landscapes [1,2]. For example, silver maple (*Acer saccharinum* L.) is a widespread native of North America and well-adapted to waterlogged soils [3]. It plays an important ecological role in the northern hardwood forests of eastern North America [4] by providing forest soils with nutrient-rich litter, promoting nitrogen mineralization and reducing nitrate leaching [5]. In Europe, these fast-growing deciduous trees with their attractive silvery leaves and tolerance to air pollution are widely cultivated in parks, streets and coastal areas [6,7,8,9]. Successful park landscaping and the creation of sustainable green spaces using silver maple requires large numbers of high-quality planting materials with healthy root systems [10]. We have previously shown that treating linden (*Tilia platyphyllos* Scop.), Scots pine (*Pinus sylvestris* L.) and black poplar (*Populus nigra* L.) seedlings with bacterial and humate preparations [11,12] accelerates the growth of both the aboveground parts of the plants and their roots. The effect of bacteria on the plant root system is explained by their ability to produce auxin phytohormones [13], which are known to stimulate root branching and seedling establishment [14]. In wheat plants treated with auxin-producing bacteria, both an increase in auxin levels and stimulation of root growth have been recorded [15]. Humic substances (HSs), which have a positive effect on the root system [16,17,18], are also capable of increasing auxin levels in plants [19]; for example, in wheat plants treated with HSs [20]. Humic acids are known to stimulate the expression of genes associated with the biosynthesis of auxins and also cytokinin hormones, which leads to increased growth of wheat roots and shoots [21]. Humic substances also exhibit auxin-like activity by activating genes associated with auxin response (e.g., *IAA5*, *IAA19*, *DR5::GUS*) and influencing signaling pathways similar to those triggered by auxin [22]. Additionally, humic acids can inhibit the activity of the enzyme IAA oxidase, which breaks down auxin [23]. However, the effect of HSs and bacteria on hormone levels in tree seedlings has not yet been studied. Of particular interest are observations that treatment of plants with a combination of HSs and bacteria stimulates root growth to a greater extent than when each is applied separately [24]. The study of the additivity of the influence of humates and auxin-producing bacteria is also promising, not only for accelerating the growth of woody plants but also for increasing carbon sequestering in soil [12]. The ability of humates to form complexes with various compounds is well known [25]. We hypothesize that the additive effect of humic substances and bacteria may be explained by the ability of HSs to bind auxins produced by bacteria; this, in turn, protects these hormones from degradation by soil enzymes and their impact on plant growth. The aim of this study was to test the ability of HSs to bind the auxin indoleacetic acid (IAA) and compare the effects of HSs and bacteria, individually and in combination, on the growth of maple seedlings and their hormone levels. Since auxin can influence the content of other phytohormones that also regulate growth by roots and shoots, the content of cytokinins and abscisic acid (ABA) in both plant roots and shoots was assessed as well [26,27,28].

## 2. Results

**Figure 1:**
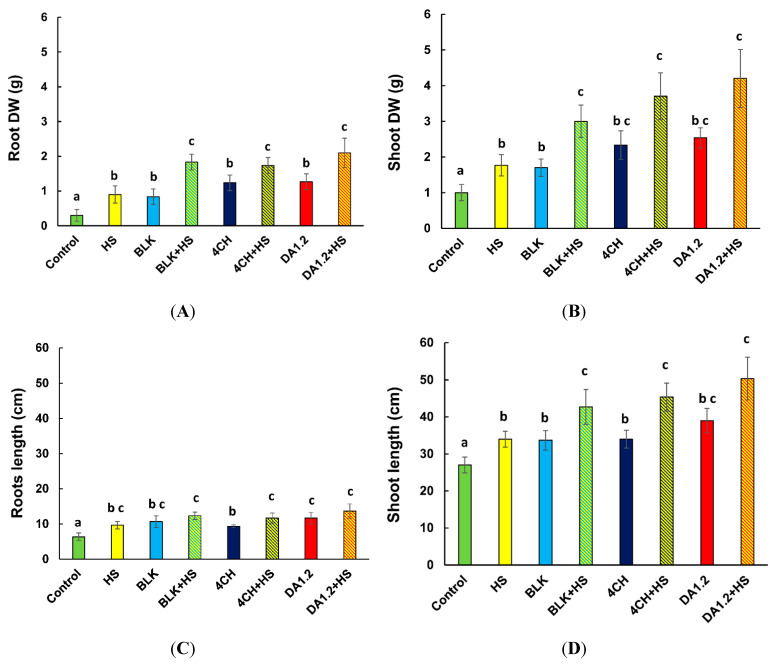
Mass (**A**,**B**) and length (**C**,**D**) of roots (**A**,**C**) and shoots (**B**,**D**) of silver maple (*Acer saccharinum* L.) after humic substances (HS), *Enterobacter ludwigii* BLK, *Pseudomonas chlororaphis* 4CH or *Pseudomonas protegens* DA1.2 were introduced into the rooting medium separately and in combination. Statistically different means are marked with different letters, *p* ≤ 0.05, *n* = 10 (ANOVA followed by Duncan’s test). Error bars correspond to SE.

The length and mass of shoots and roots increased in silver maple treated with HSs or with three species of bacteria (*Enterobacter ludwigii* BLK; *Pseudomonas chlororaphis* 4CH; *Pseudomonas protegens* DA1.2 supplied separately (Figure 1, Table S1). However, the promoting effect of HSs increased considerably when combined with any one of the three strains of bacteria used (Figure 1).

**Figure 2:**
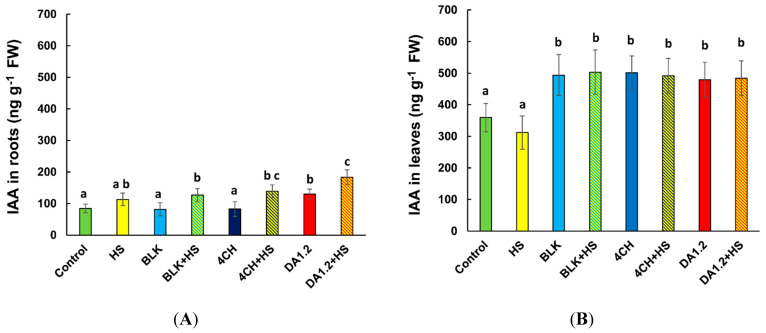
Concentration of indoleacetic acid (IAA) in the roots (**A**) and leaves (**B**) of silver maple (*Acer saccharinum* L.) after humic substances (HS), *Enterobacter ludwigii* BLK, *Pseudomonas chlororaphis* 4CH or *Pseudomonas protegens* DA1.2 were incorporated into the rooting medium separately and in combination. Statistically different means are marked with different letters, *p* ≤ 0.05, *n* = 6 (ANOVA followed by Duncan’s test). Error bars correspond to SE.

When these growth responses were compared with IAA concentrations in the roots, neither BLK nor 4CH, when added alone, increased root auxin concentration compared to the controls, and only DA1.2-treated plants had higher root IAA. When HSs and bacterial treatments were combined, increases in IAA were more conspicuous. This was especially noticeable with DA1.2 (Figure 2A). In leaves, HS treatment failed to raise IAA concentration unless combined with bacteria. Each of the bacterial strains alone stimulated IAA in the leaves. Two-way ANOVA revealed statistically significant effects of HSs on IAA concentration in roots and the bacterial effect on this hormone in leaves (Table 1).

**Figure 3:**
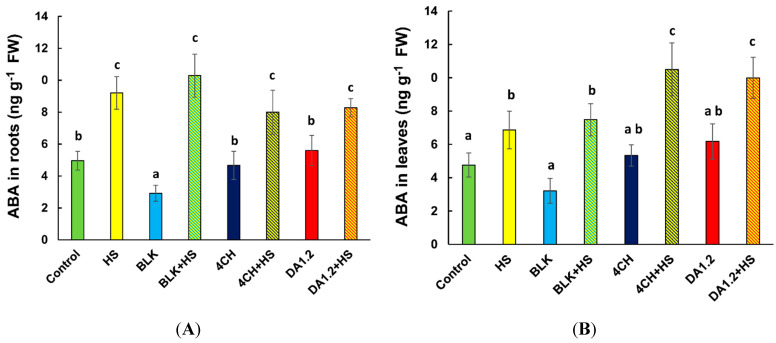
Content of abscisic acid (ABA) in the roots (**A**) and leaves (**B**) of silver maple (*Acer saccharinum* L.) after humic substances (HS), *Enterobacter ludwigii* BLK, *Pseudomonas chlororaphis* 4CH or *Pseudomonas protegens* DA1.2 were incorporated into the rooting medium separately and in combination. Statistically different means are marked with different letters, *p* ≤ 0.05, *n* = 6 (ANOVA followed by Duncan’s test). Error bars correspond to SE.

The concentration of ABA in roots was increased after treatment with HSs alone, while adding bacteria to HSs had no statistically significant additional effect (Table S1). DA1.2 and 4CH alone did not influence root ABA, although a small decrease was seen with BLK (Figure 3A). In leaves, ABA concentration was also increased by HSs alone and when combined with 4CH and DA1.2. Treatment with bacteria in the absence of HSs did not increase ABA levels in either the roots or the leaves (Figure 3B, Table S1).

**Figure 4:**
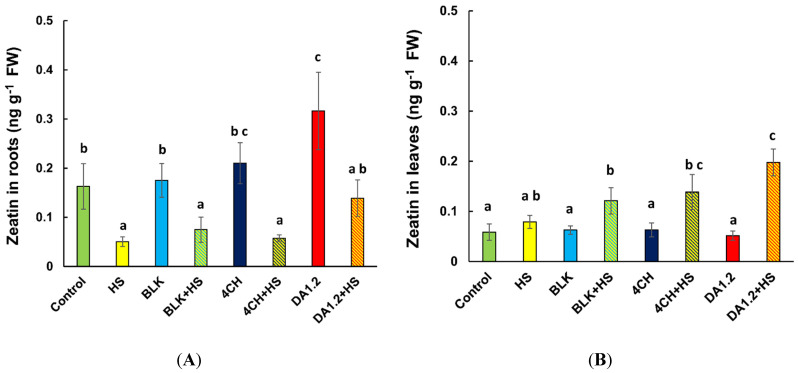
Content of zeatin in the roots (**A**) and leaves (**B**) of silver maple (*Acer saccharinum* L.) after humic substances (HS), *Enterobacter ludwigii* BLK, *Pseudomonas chlororaphis* 4CH or *Pseudomonas protegens* DA1.2 were incorporated into the rooting medium separately and in combination. Statistically different means are marked with different letters, *p* ≤ 0.05, *n* = 6 (ANOVA followed by Duncan’s test). Error bars correspond to SE.

Under HS treatment, a decrease in the concentration of zeatin (an active cytokinin) was detected in roots, both in the presence and absence of additional bacteria (Figure 4). The exception was HS treatment with DA1.2, for which no significant changes were observed compared to the control. The only bacterial treatment that stimulated zeatin accumulation in roots when given alone was DA1.2. In leaves, increased zeatin content was recorded with combined bacterial treatments and HSs, which was most pronounced for HSs with DA1.2. The trend toward increased zeatin levels with HS treatment without bacteria was not statistically significant.

**Figure 5:**
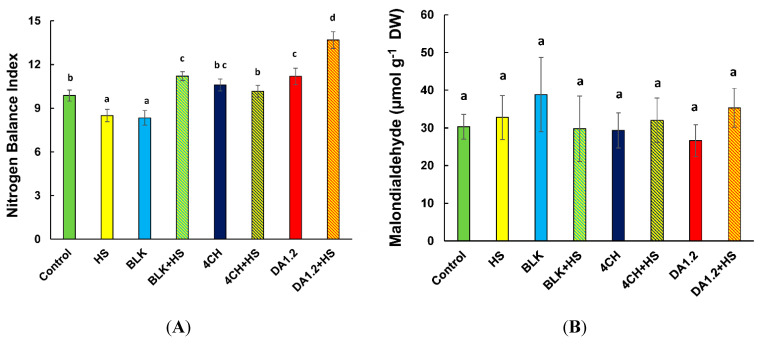
Nitrogen balance index (NBI) (*n* = 20) (**A**) and malondialdehyde content (*n* = 6) (**B**) in the leaves of silver maple (*Acer saccharinum* L.) after humic substances (HS), *Enterobacter ludwigii* BLK, *Pseudomonas chlororaphis* 4CH or *Pseudomonas protegens* DA1.2 were incorporated into the rooting medium separately and in combination. Statistically different means are marked with different letters, *p* ≤ 0.05 (ANOVA followed by Duncan’s test). Error bars correspond to SE.

The nitrogen balance index (Figure 5A) was decreased by HSs and BLK applied separately and increased by DA1.2 and a combination of BLK and HSs. The greatest increase was seen with a combination of DA1.2 and HSs. In the latter case, zeatin in leaves also increased to the greatest extent (Figure 4).

Malondialdehyde concentrations remained unchanged across all treatments, suggesting the absence of oxidative stress (Figure 5B, Table S1).

**Figure 6:**
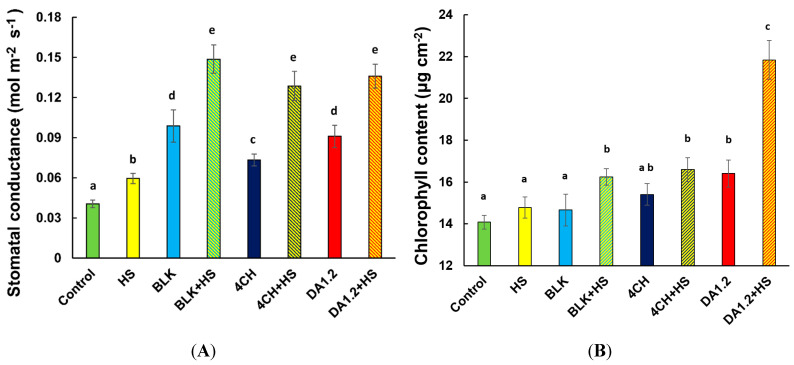
Stomatal conductance (**A**) and chlorophyll content in leaves (**B**) of silver maple (*Acer saccharinum* L.) after humic substances (HS), *Enterobacter ludwigii* BLK, *Pseudomonas chlororaphis* 4CH or *Pseudomonas protegens* DA1.2 were incorporated into the rooting medium separately and in combination. Statistically different means are marked with different letters, *p* ≤ 0.05, *n* = 20 (ANOVA followed by Duncan’s test). Error bars correspond to SE.

Stomatal apertures were influenced by HSs and bacteria, with either treatment increasing stomatal conductance (Figure 6A, Table S1). The effect of HSs was always further enhanced by the addition of bacteria (Figure 6A). In contrast, chlorophyll content on a unit area basis was little affected by HSs or bacteria, although strain DA1.2 achieved an approximately 17% increase (Figure 6B). However, the combination of HSs and bacteria always raised chlorophyll levels by a statistically significant amount (Figure 6B).

**Figure 7:**
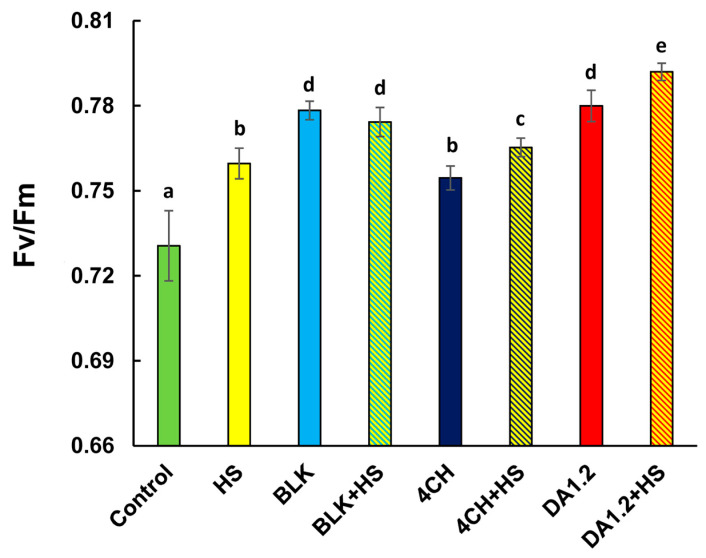
Maximum quantum yield of photosystem II (Fv/Fm) in leaves of silver maple (*Acer saccharinum* L.) after humic substances (HS), *Enterobacter ludwigii* BLK, *Pseudomonas chlororaphis* 4CH or *Pseudomonas protegens* DA1.2 were incorporated into the rooting medium separately and in combination. Statistically different means are marked with different letters, *p* ≤ 0.05, *n* = 10 (ANOVA followed by Duncan’s test). Error bars correspond to SE.

The impact on photosynthesis in terms of maximum quantum yield of photosystem II was positive for either HSs or for each of the bacterial strains given separately (Figure 7). The effectiveness of HSs was enhanced when DA1.2 and 4CH were added, with DA1.2 being the most effective strain.

**Figure 8:**
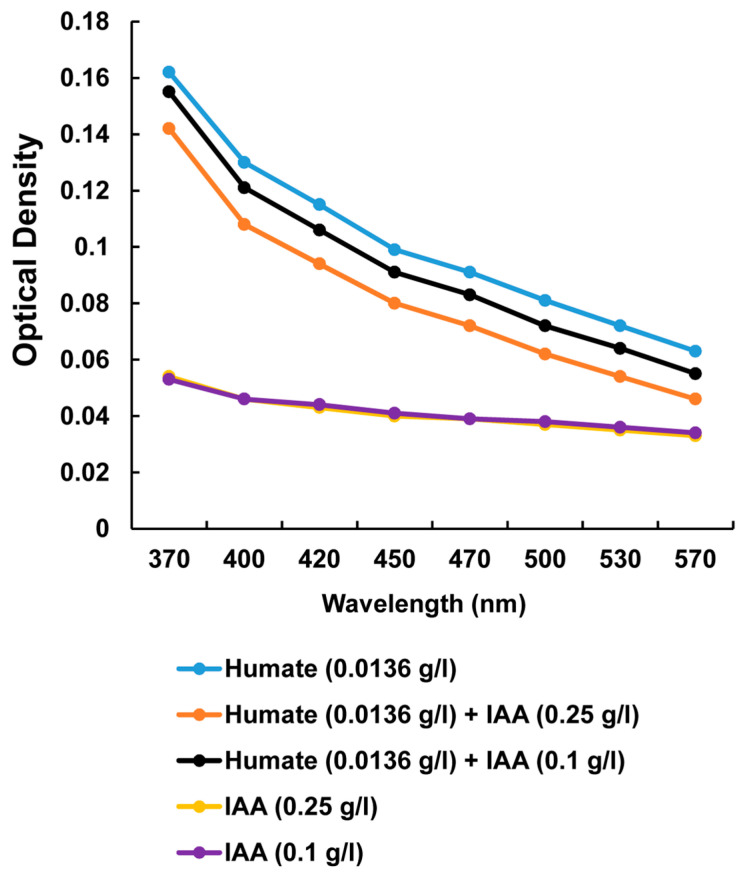
UV–visible absorption spectrum of indole acetic acid (IAA) and potassium humate (H) separately and in combination in distilled water at 20 °C: (1) H alone (0.0136 g/L); (2) IAA alone (0.1 g/L); (3) IAA alone (0.25 g/L); (4) H (0.0136 g/L) + IAA (0.1 g/L); (5) H (0.0136 g/L) + IAA (0.25 g/L).

To confirm the hypothesis that humic substances and IAA can form complexes, we determined the effect of IAA on the optical absorption spectrum of potassium humate in the 370–570 nm range when the hormone was added to humate in H_2_O. IAA itself exhibited almost no absorption in this spectral region, while humate alone was strongly absorbing (Figure 8). When IAA (0.0136 g/L) was added to humate, its optical density reading was depressed, and the depression increased further in the presence of a higher IAA concentration (0.25 g/L) (Figure 8). This is explained by the formation of a complex between IAA and potassium humate (H) with an optical density lower than that of the humate itself.

**Figure 9:**
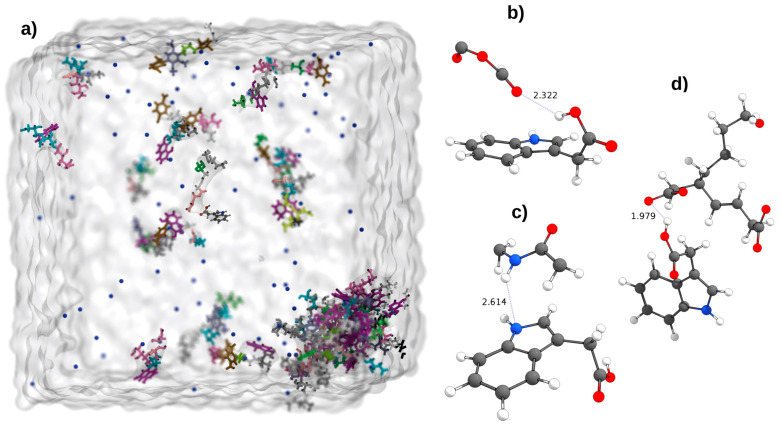
Molecular dynamics simulation of the interaction of HSs with indole acetic acid (IAA). (**a**) General scheme of the system. IAA is located in the center; the fragment colors are intended only for visual separation of individual humic fragments; (**b**) typical frame of contact of IAA with fragment HS11; (**c**) with fragment HS18; and (**d**) with fragment HS3. Black spheres—carbon, red—oxygen, blue—nitrogen, white—hydrogen. Snapshots in panels (**b**–**d**) show key hydrogen-bond distances (Å).

Molecular dynamics modeling of the interaction of HSs with IAA allowed us to analyze the overall intensity of the contacts between the two types of molecules. This approach indicated that the interaction between IAA and HSs is significantly heterogeneous and is determined by a relatively small number of the most active fragments (Figure 9). Individual fragments stand out in the humic substrate and can be considered local “hot spots” of interaction. These fragments are the main contributors to hormone sorption and retention. The strongest interaction was observed for the fragment denominated as HS11. Its contact matrix demonstrates a high intensity of contacts simultaneously for several atomic pairs: N–O1, N–O2, O1–O1, O1–O2, O2–O1, and O2–O2 (explanation of abbreviations can be found in Section 4 and Supplementary Materials). This spectrum of contacts clearly indicates the leading role of oxygen-containing functional groups of the humic fragment. It is important to note that the predominance of O-containing contacts does not imply a complete absence of the contribution of other types of interactions. However, it is the oxygen functional groups that form the main ensemble of nearby intermolecular contacts that determine the retention of IAA in the system we studied. The most significant maxima of the radial distribution functions are located predominantly in the range of 2.5–3.2 Å, i.e., in the range characteristic of hydrogen bonds and strong polar contacts. Less intense interactions are observed in the range of 3.2–4.0 Å, corresponding to weaker polar or short-range Van der Waals contacts. Thus, directional and relatively short-range polar contacts play a leading role in the interaction of IAA with humic acid.

## 3. Discussion

Treatments with bacteria and HSs, separately and in combination, stimulated maple plant growth in terms of shoot and root length and fresh mass. The effect of the three bacterial preparations is linked to increased levels of auxin IAA in leaves. This is not surprising, since these bacteria are capable of producing auxins [12,29]. The ability of auxins to stimulate shoot growth is known [30], and IAA concentration was higher in the leaves of plants treated with all three bacterial strains (*Enterobacter ludwigii* BLK; *Pseudomonas chlororaphis* 4CH; *Pseudomonas protegens* DA1.2). This was the case when they were applied both in combination with HSs and without them. Adding HSs to bacteria did not affect IAA levels in maple leaves, indicating that the increased auxin content was due to bacteria, not the HSs. However, HSs did contribute to the stimulation of shoot growth since shoot mass and length were greater in plants treated with HSs compared to the control. Furthermore, the combination of HSs and bacteria led to a greater growth-promoting effect compared to the use of bacteria alone. We propose the following explanation for the action of HSs as a shoot growth stimulator. Treatment with HSs, both alone and in combination with bacteria, increased root IAA, and this, in turn, stimulated root growth (a known effect of IAA) [14,30]. It is notable that no increase in auxin was detected in roots of plants treated with BLK and 4CH bacterial strains without HSs. The absence of an increase in root IAA content in plants treated with these bacteria capable of synthesizing auxins suggests that activated IAA flowed into the leaves of the plants, where elevated levels of auxins were observed. Effects of humates on auxin levels in leaves can be explained by the presence of these hormones in preparations of humic substances [31]. Thus, stimulation of root growth under the influence of HS-containing preparations is explained by their ability to raise auxin levels in roots.

Most intriguing is the increase in auxin levels in the roots when HSs are combined with bacteria capable of synthesizing auxins. We hypothesized that this effect may be related to the ability of HSs to bind bacterial auxins entering the soil and deliver them to plants. It is known that HSs can bind different substances and deliver them to the root surface [32]. Some bacteria used in the present work are characterized as endophytic [33]. However, they are also present in the plant rhizosphere and colonize the root surface [34,35]. Thus, interactions between humic substances and bacterial hormones in soil are highly probable.

An assessment of the UV–visible absorption spectra of HSs and IAA revealed changes upon the addition of IAA to HSs, suggesting the formation of a complex between them. Formation of this complex was also confirmed with molecular dynamics modeling and analysis of the humic acid–indole-3-acetic acid system using the Soil Organic Matter Model platform. This revealed a fragment designated HS11 with the strongest interaction with IAA. Its contact matrix demonstrates the leading role of oxygen-containing functional groups of the humic fragment and that the nitrogen atom of IAA is preferentially located near the carbonyl or hydroxyl/ester oxygen atoms of HS11. The participation of the oxygen atoms of IAA itself in polar binding to the oxygen-containing centers of humic acid was also revealed. Chemically, this pattern is characteristic of a fragment rich in carboxyl and phenolic groups. These groups are capable of creating a dense network of polar and hydrogen bonds with the carboxyl moiety of IAA, as well as stabilizing the molecule’s association with its immediate environment. Thus, HS11 can be considered the most prominent binding fragment in the studied system. The binding of auxins such as IAA to HSs protects this labile hormone from degradation by soil enzymes, which can explain increased auxin content in plant roots treated with a combination of bacteria and humates.

Molecular dynamics modeling indicates that the interaction of IAA with humic substances is determined not by the average composition of the humic matrix but by the presence of individual structural fragments capable of providing a tight polar environment for the hormone molecule. This means that sorption and retention of the phytohormone in the soil organic matrix can be localized to a relatively small number of structural motifs. From a practical perspective, this is important for understanding the mobility, bioavailability, and stability of the hormone in natural and man-made systems. Furthermore, the leading role of the identified oxygen-containing functional groups suggests that changes in pH, ionic strength, and the degree of protonation of humic centers will significantly influence the nature and extent of IAA binding. Therefore, it is likely that acidification of the rhizosphere due to root exudation [36] can influence stability of the IAA/HSs complex and lead to its dissociation, thereby releasing unbound hormone.

Interestingly, we did not observe auxin accumulation in roots across all bacterial treatments, although all of them stimulated root growth. Neither BLK nor 4CH increased root auxins compared to the control, and only DA1.2-treated plants had higher root IAA concentrations than the control. In the absence of IAA accumulation in roots, stimulation of root growth can be explained by an increased influx from shoots of substrate for root growth as a result of activated photosynthesis.

Assessment of zeatin content in plant leaves revealed increased levels of this active form of cytokinin in plants treated with humates in combination with bacteria. This effect was most pronounced in the treatment combining humates with the DA1.2 bacteria strain and was associated with increased chlorophyll and nitrogen index levels in plants. This link was most noticeable when humates were combined with the DA1.2 strain, which yielded the highest zeatin content in leaves. The ability of cytokinins to stimulate photosynthesis and shoot growth is well known [37]. Cytokinins also contribute to the maintenance of chlorophyll levels in plants [38]. The ability of humates to stimulate cytokinin accumulation in plants has also been demonstrated [39]. The effect of humates on cytokinins is explained both by the presence of this hormone in the HS preparation [40] and by the ability of humates to modify cytokinin production by plants themselves [41]. In seedlings treated with humic acid, increased expression of several genes involved in cytokinin biosynthetic pathways (*Lonely Guy3*) was observed [21]. However, in our experiments, zeatin accumulation in the leaves of plants treated with HS-containing preparations occurred against a background of decreased levels of zeatin in the roots. These results suggest that zeatin accumulation in the leaves may be a consequence of altered distribution of this hormone from the roots to the leaves. This hypothesis is consistent with published data showing that humic acid-induced cytokinin accumulation in cucumber leaves and stems occurred against a background of decreased levels of these hormones in the roots [42]. Thus, the ability of HSs to influence cytokinins could have contributed to increased photosynthetic pigment levels, photosynthesis, and, ultimately, accelerated growth in the maple plants.

Many of the treatments used in our experiments maintained stomata in the open state (increased stomatal conductance compared to the control), which was most noticeable when plants were treated with a combination of bacteria and HSs. Thus, the increase in stomatal conductance under the influence of the various treatments could have contributed to the increased photosynthesis rates recorded in these experiments by facilitating CO_2_ uptake. The ability of ABA to regulate stomatal conductance by inducing stomatal closure is well known [43,44]. However, it was not possible to link the effect of treatments on stomata with their effect on ABA levels since they either failed to affect ABA levels in leaves or increased them. Stomatal conductance is influenced not only by ABA but also by cytokinins, which, unlike ABA, maintain stomata in an open state [45]. Accordingly, stomatal conductance was highest for the treatments that induced the most significant increases in zeatin levels.

However, ABA may also have indirectly contributed to the increase in stomatal conductance. This hormone can have a dual effect on stomatal conductance, not only closing stomata when it accumulates in leaves, but also maintaining them open through its effect on root hydraulic conductance [46]. The latter effect is due to an ABA-induced increase in aquaporin activity [47]. Its accumulation in the roots leads to increased water influx from the roots, maintaining stomata open. Inoculation of wheat plants with *Bacillus subtilis* IB22 bacteria reduced ABA levels in shoots and led to ABA accumulation in the roots [48], which increased transpiration. In our experiments, HSs increased ABA levels in the roots, which apparently maintained increased stomatal conductance, gas exchange, and photosynthesis indirectly, thereby promoting maple plant growth.

ABA accumulation is often observed in plants exposed to stress [49]. Another indicator of stress is an increase in MDA concentration as a result of membrane damage in stressed plants [50]. However, in the present experiments, no increase in MDA content was detected, indicating the absence of stress. These results suggest that the increase in ABA levels in our case was caused not by stress but by another signal. Olaetchea et al. found that treating cucumber with humic acids increased ABA concentrations in plants, accompanied by an increase in hydraulic conductivity and aquaporin gene expression [51], suggesting that humic acids may be involved in the signal-mediated increase in ABA levels.

Thus, the additional effect of humates on bacteria-promoted growth can be explained by the positive effect of humates on root growth due to an increase in auxin levels and on shoot growth due to an increase in stomatal conductance and chlorophyll content. This complements the effect of bacteria that increases auxin levels in the leaves. Molecular dynamics modeling and the study of the UV–visible spectral characteristics of HSs in the presence of IAA revealed the ability of HSs to bind to this auxin, which helps maintain its level in soil and plants. The combination of HSs with bacterial preparations also affected ABA and zeatin levels in plants, which may also be related to the ability of HSs to bind not only auxins but also other hormones. This possibility now requires verification. The additive effect of HSs and auxin-producing bacteria could usefully be harnessed to formulate preparations able to stimulate the growth of woody plants such as sugar maple in planting schemes designed to regenerate challenging landscapes, promote carbon sequestration and help mitigate anthropogenic climate change.

## 4. Materials and Methods

### 4.1. Plant Growth Conditions and Treatments

The effects of the humic substances and bacterial preparations were studied on 2-year-old silver maple (*Acer saccharinum* L.) seedlings (Figure S1). The plants were grown from freshly picked seeds collected from silver maple at the South Ural Botanical Garden-Institute, Ufa. Fruiting is typically sporadic, and abundant seed harvests are rare, making it especially important that seedlings raised for landscape plantings establish themselves successfully. The experiments were conducted at the South Ural Botanical Garden-Institute and utilized standard technology for growing tree seedlings in nurseries (national “Green” standards of the Russian Federation, 2021) [52]. Freshly harvested seeds were sown in June 2024. One seedling was planted in each 1 L container (15 × 15 × 15 cm) on 15 April 2025. The soil mixture comprised black soil from an ecologically clean area, manure from a stud farm and sterilized river sand in the ratios of 3:1:1. The seedlings were watered regularly. At the time of planting, 100 mL of one of three species of bacteria in suspension (4 ± 0.5 × 10^9^ CFU/mL) and humic acid (HSs, 2 g/L) were added to the potting soil mixture, either separately or in combination. The application rate of HSs was based on previous experiments that confirmed its effectiveness as a growth stimulant [11]. The same treatments were given the following week and repeated once a month for four months. Control plants were treated with the same amount of water without additives. There were 20 pots with one plant each per treatment. Non-destructive measurements of chlorophyll, NBI, and stomatal conductance were taken for all plants in each treatment two weeks before the end of the experiments. Samples for hormone and MDA analysis, as well as shoot and root weight and length measurements, were collected at the end of the experiments.

### 4.2. Bacterial Strain and Humic Substances

The three strains of Gram-negative bacteria used from the micro-organism collection of the Ufa Institute of Biology were *Pseudomonas protegens* DA1.2 (deposited in the All-Russian Collection of Microorganisms as B-3542D); *Pseudomonas chlororaphis* 4CH (deposited as UIB-57) and *Enterobacter ludwigi* BLK (deposited as B-3652D). Bacteria of the DA1-2, 4CH and BLK strains produce IAA in quantities of approximately 900, 800 and 2000 ng/mL of culture fluid, respectively [53]. These bacterial strains were chosen after previous experiments showed their ability to stimulate the growth of seedlings of Scots pine (*Pinus sylvestris* L.), poplar (*Populus italica pyralis × P. nigra*), large-leaved linden (*Tilia platyphyllos* Scop.) and horse chestnut (*Aesculus hippocastanum* L.) [12,53] in combination with HSs. The bacteria were cultured in Erlenmeyer flasks (MiniMed, Bryansk, Russia) with King’s B medium (2% peptone, 1% glycerol, 0.15% K_2_HPO_4_, 0.15% M H_2_SO_4_) on an Innova 40R shaker (New Brunswick, NJ, USA) at 160 rpm for 48 h at 28 °C [53].

Brown coal (Tyulganskoye deposit, Orenburg region, Russian Federation) was used as a source of humic substances. The coal was mixed with 0.1 M KOH in a ratio of 1:10, and the HSs were extracted over 24 h with mixing at 1500 rpm. The precipitate was removed by centrifugation at 12,000 rpm for 10 min [12]. The HSs used in the present study have been characterized previously by ^1^H NMR, ^13^C NMR, IR, UV spectroscopy, and UV spectrofluorimetry [54]. The characteristics of the HSs are summarized as follows: The ^13^C NMR spectra reveal keto groups and aromatic carbon associated with O and bound to aliphatic carbon. In the ^1^H NMR spectra, peaks were found in the range of C, as well as H-substituted aliphatic fragments, aliphatic fragments in position α to an electronegative group or aromatic ring, aliphatic fragments doubly substituted with heteroatoms and CH-substituted aromatic fragments. IR spectra indicated the presence of hydroxyl groups (phenolic, alcohol, and OH groups in carboxyl groups), long methylene chains, methyl end groups, carboxyl groups and benzoid structures in aromatic systems. UV–visible spectra of sodium humates (0.08% solution of humic acid (HA) in 0.05 N NaOH) exhibited absorption bands at E^465^ = 3.42; E^665^ = 0.789, which correspond to the literature data for humic substances [55,56], including sodium fulvates (0.10% solution of fulvic acids (FAs) in 0.05 N NaOH): E^465^ = 0.109; E^665^ = 0.096 and for 0.001% solution of fulvates: E^465^ = 0.0011; E^665^ = 0.00036. E^465^/E^665^ = 3.3. Fluorescence spectra of humates contained emission peaks (λ max) at 310 nm (0.081% in 0.05% NaOH) and 418 nm. Emissions at 310 nm (0.10% in 0.05 NaOH) and 421 nm corresponded to either HA or FA [57]. A more detailed description by Feoktistova et al. is available [54].

### 4.3. Extraction of IAA and ABA and Their Immunoassay

The hormones ABA, IAA, and cytokinins were extracted from crushed leaves and roots with 80% ethanol according to [53,58,59]. After evaporation of alcohol, the aqueous residue was centrifuged, and the supernatant was collected for further purification. Purification of ABA and IAA was carried out using a scheme based on decreasing the volumes of extractant at each stage. For this, the aqueous residue was adjusted to pH 2.5 with HCl and extracted twice with diethyl ether at an organic to aqueous phase ratio of 1:3. IAA and ABA were then transferred from diethyl ether to sodium bicarbonate at a 1:3 aqueous to organic phase ratio. The pH was adjusted to 2.5 and the hormones re-extracted with ether. Decreasing the volume of extractants at each stage ensured the selectivity of hormone extraction. IAA and ABA were then methylated with diazomethane to enhance cross-reactivity with antibodies. To perform the enzyme-linked immunosorbent assay (ELISA), hormone–ovalbumin conjugate dissolved in phosphate buffer was adsorbed onto the well walls of a microplate. After washing the plate three times with 100 mM NaCl containing phosphate buffer, Tween 20 and ovalbumin, a mixture of various concentrations of hormone standard or sample was incubated with antihormonal rabbit serum. The method is based on competition for antibodies between sample hormones and a hormone–protein conjugate adsorbed on the sorbent walls. After washing off unbound rabbit serum, secondary antibodies against rabbit immunoglobulins conjugated with peroxidase were incubated in the wells. After a further wash, the peroxidase substrate (o-phenylenediamine in phosphate buffer pH 5.5 with 3% H_2_O_2_) was added. The light absorption by the reaction product was measured using a microphotometer (Uniplan, Moscow, Russia). The reliability of the immunoassay of ABA and IAA was confirmed by comparison of the results obtained with the immunoassay with those of a physicochemical assay [53,59].

Cytokinins from the aqueous residue were concentrated using a pre-wetted C18 column (Waters, Milford, MA, USA). After washing with 20 mL of distilled water loaded onto the column, cytokinins were eluted with 5 mL of 80% ethanol. After evaporation of the solvent, the dry residue was dissolved in 0.02 mL of 80% ethanol and subjected to thin-layer chromatography (TLC) [58]. Cytokinins were separated on Merck silica gel 60 F-254 plates with a mixture of 2-butanol: 14 M NH_4_OH: H_2_O (6:1:2 by volume). TLC zones containing cytokinins were identified in relation to position of zeatin. The zones were eluted with 0.1 M phosphate buffer at pH 7.4 for 12 h; the eluents were added directly to the wells of the microplate in several dilutions for immunoassay, as described by Kudoyarova et al. [58]; and an ELISA was performed using zeatin-specific antibodies [58].

### 4.4. Stomatal Conductance, Chlorophyll Content and Fluorescence

Stomatal conductance was measured using an LI-600 porometer/fluorometer (LI-COR Biosciences, Lincoln, NE, USA). Chlorophyll and flavonoid content in leaves was determined, and the nitrogen balance index (NBI) was calculated using a DUALEX SCIENTIFIC+ instrument (FORCE-A, Paris, France). Chlorophyll fluorescence of intact leaves was measured using a Junior PAM fluorometer (Walz, Germany) using WinControl 3 software version 3.25. Before measurements, plants were kept in the dark for 30 min. The maximum photochemical quantum yield of Photosystem 2 was calculated using the formula Fv/Fm = (Fm − F0)/Fm), where Fm is the maximum chlorophyll fluorescence yield in dark-adapted leaves in response to a saturating flash of light, and F0 is the minimum chlorophyll fluorescence yield under conditions that do not cause changes in the state of the photosynthetic apparatus in dark-adapted leaves.

### 4.5. Malondialdehyde Assay for Lipid Peroxidation

Lipid peroxidation intensity was tested by measuring MDA using a color reaction with 2-thiobarbituric acid, followed by measurement of optical density at wavelengths of 532 nm and 600 nm [60]. The concentration of MDA (A) was calculated using the formula A = ((D_532_ − D_600_) × 10 × V × L)/(P × E), where D532 and D_600_ are optical densities at 532 nm and 600 nm, respectively; V = volume of the reaction mixture; L = ratio of the total volume of the extract to the volume of the sample; E = molar extinction coefficient (155,000 1/(cm mol); and P = mass of the plant material (g fresh weight).

### 4.6. Method for Studying the UV–Visible Spectra of Humates in the Presence of IAA

A solution of humate was used (the production procedure is described above) in distilled water. Different concentrations of IAA (Sigma-Aldrich, Shanghai, China) were added to humate (0.0136 g/L) in H_2_O. The UV–visible spectra of IAA, humate and their mixture were registered with a UV-2600 spectrophotometer (Shimadzu, Kyoto, Japan) at wavelengths of 350–570 nm.

A system developed using the Soil Organic Matter Model (SOMM, University of Natural Resources and Life Sciences, Vienna, BOKU) platform was used as a full-atom model of humic acid. The Elliott IV humic acid system, constructed based on solid-phase 13C NMR spectroscopy data and available in the SOMM library (https://somm.boku.ac.at/; https://somm.boku.ac.at/eq_systems/elliottIV_HA_SSNMR.tar.gz (accessed on 25 April 2026), was chosen for modeling. The humic substance was considered as a set of individual fragments, HS1–HS29. This approach allows us to move from an average description of humic acid to fragment-specific analysis and determine which structural elements of the humic substance are most involved in the interaction with the hormone molecule under study. Indoleacetic acid (IAA) was considered in its neutral form.

Molecular dynamics calculations were performed using the Desmond package version.7.2.134. The system was a model of humic acid with an IAA molecule in an explicit aqueous environment within a periodic cell. Long-range electrostatic interactions were accounted for using the standard particle mesh Ewald (PME) method, while short-range noncovalent interactions were calculated using a standard cutoff scheme. Equilibrium was achieved in the NPT ensemble at 300 Kelvin temperature and atmospheric pressure. After a relaxation step, the main simulation was run for approximately 100 ns.

For analysis, the atoms of both the hormone and humic acid fragments were divided into three types according to their chemical environment: N—nitrogen atoms; O1—oxygen atoms with one covalent bond (e.g., carbonyl oxygen); O2—oxygen atoms with two covalent bonds (e.g., hydroxyl or ether oxygen). For each humic acid fragment and for each of the nine combinations of atomic types (N–N, N–O1, N–O2, O1–N, O1–O1, O1–O2, O2–N, O2–O1, O2–O2), the radial distribution function g(r) was calculated. To identify significant intermolecular contacts, the analysis was limited to a range of distances no greater than 4 Å. This range encompasses typical hydrogen bonds (approximately 2.5–3.2 Å), strong polar contacts (approximately 3.0–3.5 Å), and short Van der Waals interactions (up to ~4.0 Å). For each g(r) function, the integrated contact intensity was calculated as a numerical characteristic of the interaction strength and/or frequency. Details of this approach and results are presented in Supplementary Materials (Table S2 and Figures S2–S18).

### 4.7. Statistics

The data were processed using two-way analysis of variance (ANOVA) to reveal the effects of HSs and bacterial treatments on plant characteristics, and one-way ANOVA and Duncan’s multiple comparison test were used to identify differences between mean values (*p* < 0.05) using Statistica version 10 (Statsoft, Moscow, Russia). Data in figures and tables are presented as mean values ± standard error. In the figures, mean values that are statistically different from each other are indicated by different letters. The number of biological replicates (n) is provided in the figure’s legends.

## Figures and Tables

**Table 1:** Two-way ANOVA test of variables between groups: control plants untreated with either bacteria or HSs, and plants treated with HSs, bacteria (BLK, 4CH and DA1.2) and their combinations performed to determine significant effects of HSs and bacterial treatments. Values of *p* in the cells corresponding to significant effect of factor are marked in red.

Variables	Source of Variation	Sum of Squares	Degree of Freedom	Mean Square	F	Sig. (*p*)
**Root IAA**	HSs	570.375	1	570.375	0.054853	0.817795
	Bacteria	113,176.1	3	37,725.38	3.628036	0.035995
	Interaction	6139.458	3	2046.486	0.19681	0.897027
**Shoot IAA**	HSs	10,427.46	1	10,427.46	**5.91555**	0.027122
	Bacteria	4880.533	3	1626.844	0.922917	0.452184
	Interaction	1345.722	3	448.5739	0.254478	0.857008

## Data Availability

The original contributions presented in this study are included in the article/Supplementary Materials. Further inquiries can be directed to the corresponding author.

## Supplementary Materials

The following supporting information can be downloaded at: https://www.mdpi.com/article/10.3390/ijms27125494/s1.

## Abbreviations

The following abbreviations are used in this manuscript:

HSs	Humic substances
ABA	Abscisic acid
IAA	Indole-3-acetic acid
NBI	Nitrogen balance index
ELISA	Enzyme-linked immunosorbent assay
SOMM	Soil organic matter model
PME	Particle mesh Ewald
