# Supplementary material for: The Influence of Humic Substances and Auxin-Producing Bacteria on Acer saccharinum Plants in Relation to Auxin-Humate Binding
Source: Int J Mol Sci. 2026 Jun 18;27(12):5494. doi: 10.3390/ijms27125494 (PMC13299725; doi:10.3390/ijms27125494)
Supplement: Supplementary file 1 [file ijms-27-05494-s001.zip › ijms-4328882-supplemntary.pdf]

Supplementary Materials

Atom-pair contact matrix and RDF analysis of IAA-humic acid interactions

This supplementary file provides the quantitative contact-matrix heatmaps and per-fragment radial distribution function (RDF, g(r)) plots requested to strengthen the molecular interpretation of the interaction between indole-3-acetic acid (IAA) and humic acid fragments.

1. Analysis summary

The humic substrate was represented by discrete molecular fragments denoted as HSx. The hormone molecule was indole-3-acetic acid (IAA), treated as a neutral molecule. Atom-pair RDFs were calculated for atom-type combinations between IAA and each humic fragment. The atom types were defined as N (nitrogen atoms), O1 (oxygen atoms with one covalent bond, e.g., carbonyl oxygen), and O2 (oxygen atoms with two covalent bonds, e.g., hydroxyl or ether oxygen).

The RDFs were calculated for all available atom-pair combinations using a distance range relevant to intermolecular contacts. Contact intensity was evaluated as the RDF integral over the short-range contact region, 0-4 Å. This metric reflects the relative frequency and intensity of close contacts between the selected atom types. The r_peak heatmap reports the position of the RDF maximum for each atom-pair interaction, whereas the contact-intensity heatmap reports the integral of g(r) within 4 Å.

The RDF plots in this file show the short-range region from 1 to 5 Å for each humic fragment. This range was selected because it contains hydrogen-bond distances, strong polar contacts, and short van der Waals contacts relevant to IAA retention by humic matter.

S2. Total contact strength of humic fragments

Table S1 summarizes the total contact strength calculated by summing the contact integrals over all atom-pair combinations for each humic fragment. The strongest contributors are HS11, HS18, and HS3, followed by HS29 and HS26.

Rank	Humic fragment	Total contact strength
1	HS11	1611.241
2	HS18	1020.432
3	HS3	589.222
4	HS29	456.274
5	HS26	417.527
6	HS13	258.058
7	HS25	166.145
8	HS22	156.885
9	HS24	137.095
10	HS6	74.704
11	HS9	50.954
12	HS28	18.832
13	HS20	8.383

Table S1. Total contact strength of IAA-humic acid fragment interactions calculated from atom-pair RDF contact integrals within 4 Å.

S3. Supplementary contact-matrix heatmaps

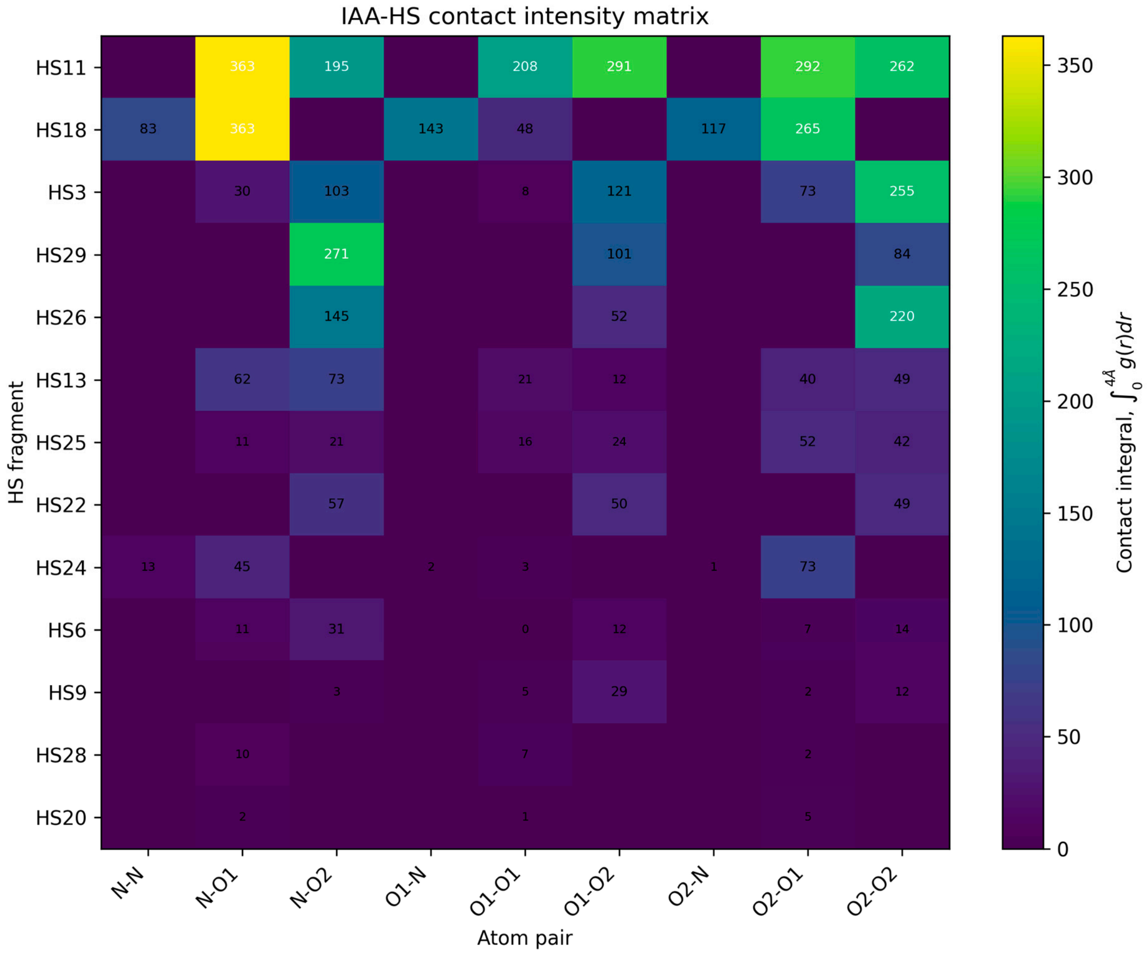

Figure S1. Contact-intensity heatmap for atom-pair interactions between IAA and humic acid fragments. The values correspond to the integrated short-range RDF intensity, ∫0-4 Å g(r)dr. Higher values indicate more frequent and/or more intense short-range contacts. The map highlights the dominant contribution of oxygen-containing functional groups, especially for HS11, HS18, and HS3.

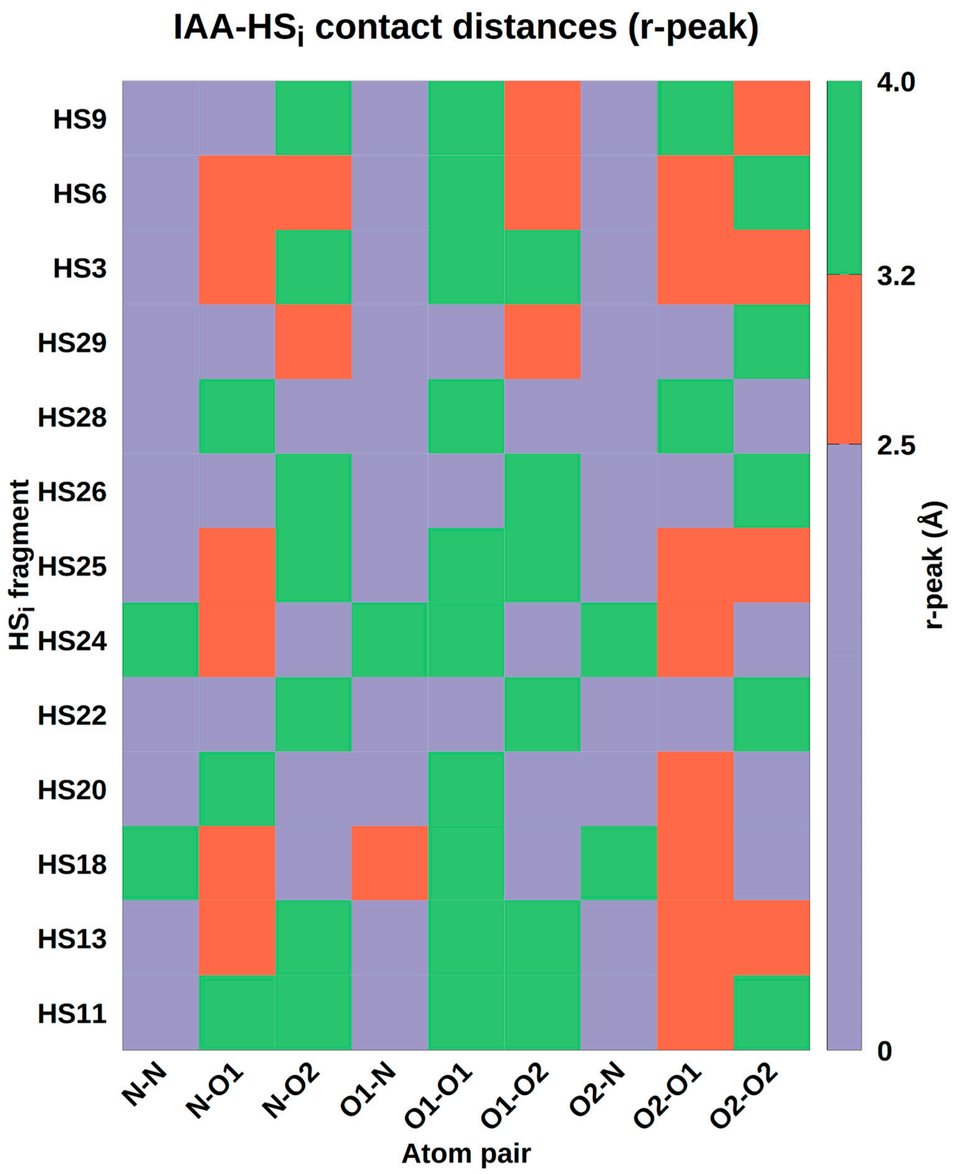

Figure S2. RDF peak-position heatmap for atom-pair interactions between IAA and humic acid fragments. The map reports r_peak values in Å and distinguishes the main distance regimes: 2.5-3.2 Å, corresponding to hydrogen bonds and strong polar contacts, and 3.2-4.0 Å, corresponding to weaker polar or short-range van der Waals contacts.

S4. Atom-pair RDF profiles for individual humic fragments

Figures S3–S17 provide the underlying g(r) profiles for individual humic fragments. Each plot contains all available atom-pair RDF curves for the corresponding fragment. All plots use the same x-axis range (1–5 Å), which facilitates comparison of short-range contact features across the humic fragments. For fragments HS7 and HS1 (Figures S16 and S17), no short-range atom-pair contacts satisfying the 4 Å cutoff were detected during the analyzed trajectory. Therefore, the corresponding RDF panels do not contain visible contact peaks and are retained in the Supplementary Materials to document the absence of detectable IAA–fragment contacts under the applied contact criterion.

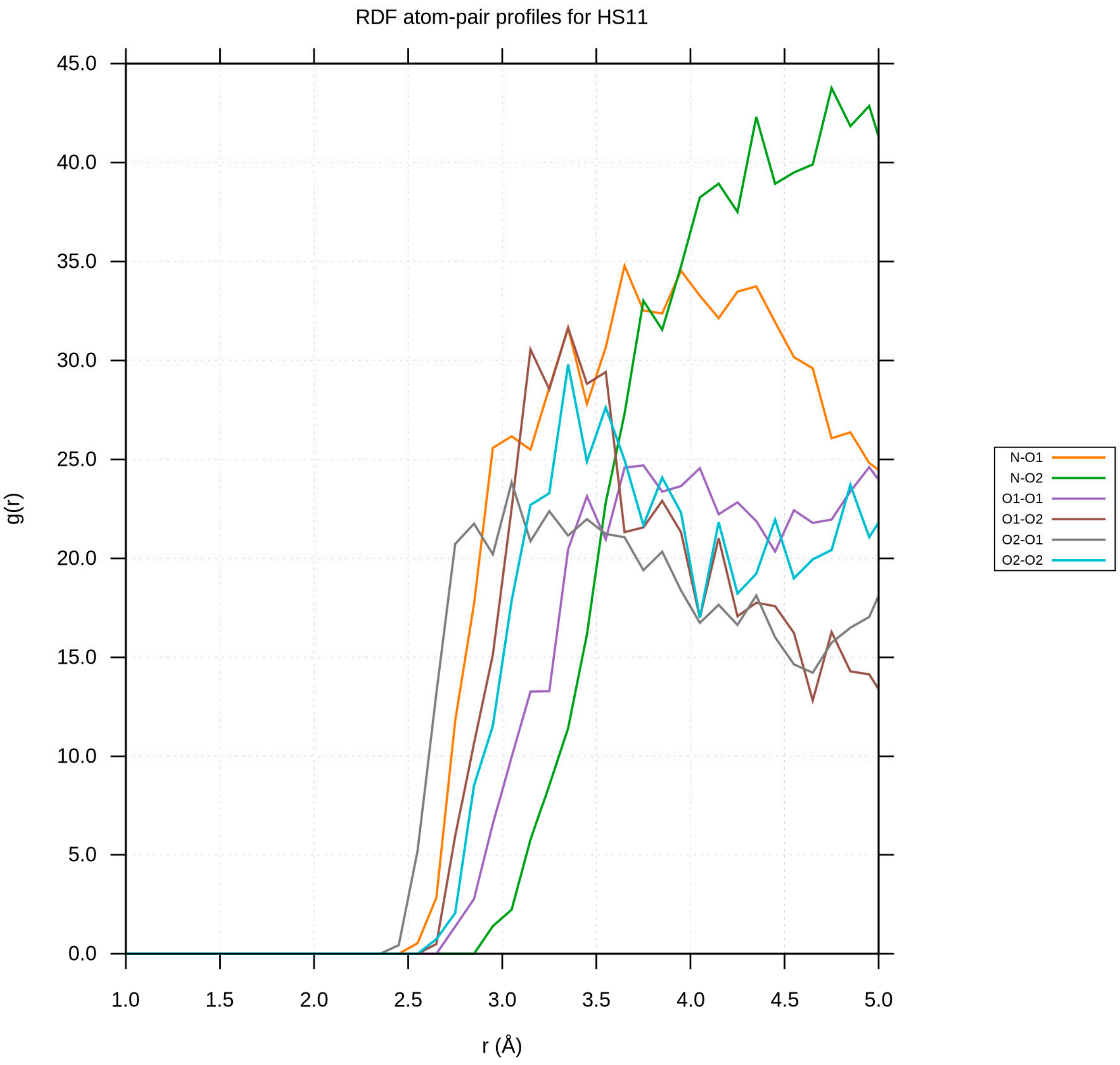

Figure S3. Atom-pair RDF profiles g(r) for IAA interactions with humic fragment HS11. Curves correspond to the available atom-type combinations involving N, O1, and O2 atoms. The displayed region (1-5 Å) covers the short-range contact domain relevant to hydrogen bonding and polar interactions. This fragment is among the three most active contributors to IAA binding.

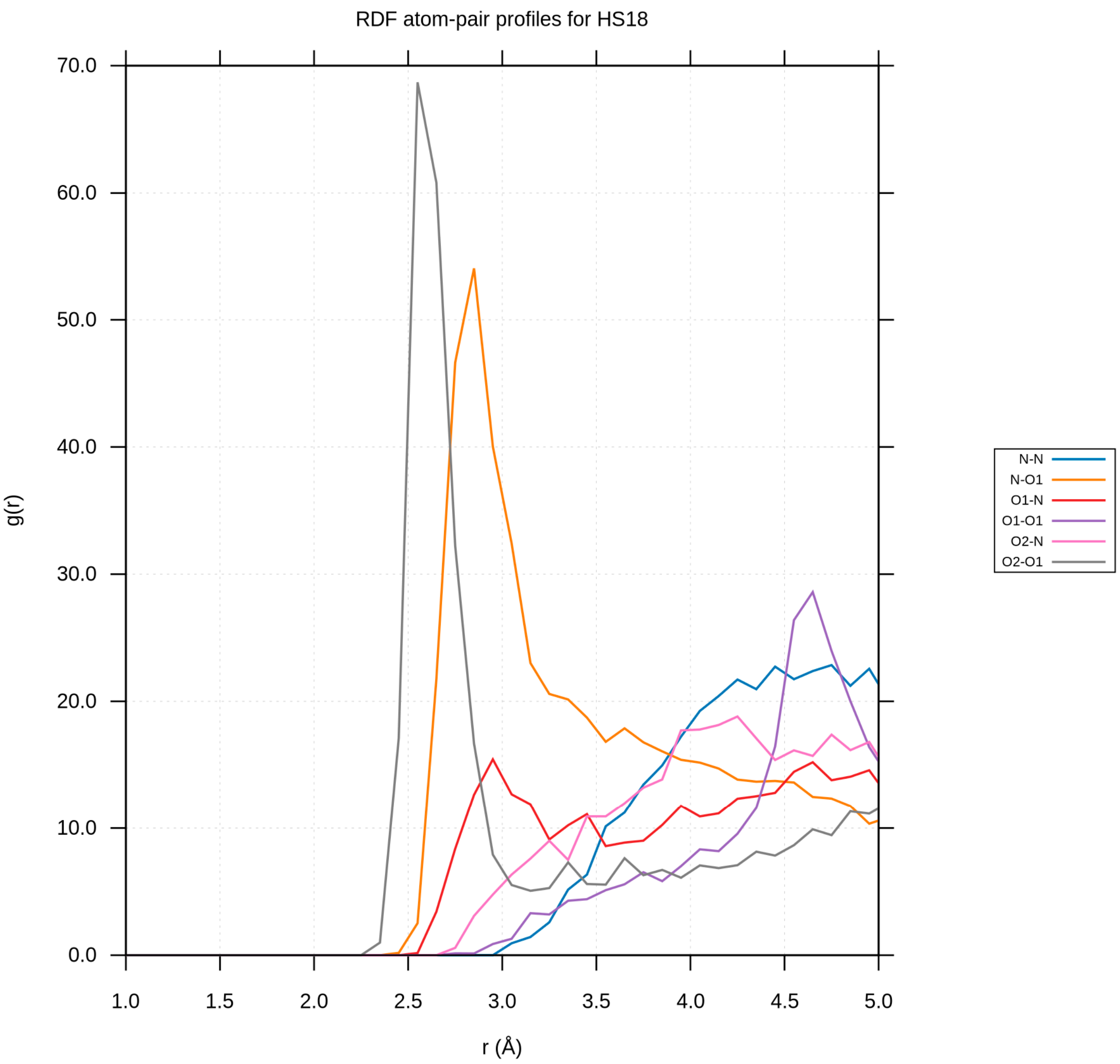

Figure S4. Atom-pair RDF profiles g(r) for IAA interactions with humic fragment HS18. Curves correspond to the available atom-type combinations involving N, O1, and O2 atoms. The displayed region (1-5 Å) covers the short-range contact domain relevant to hydrogen bonding and polar interactions. This fragment is among the three most active contributors to IAA binding.

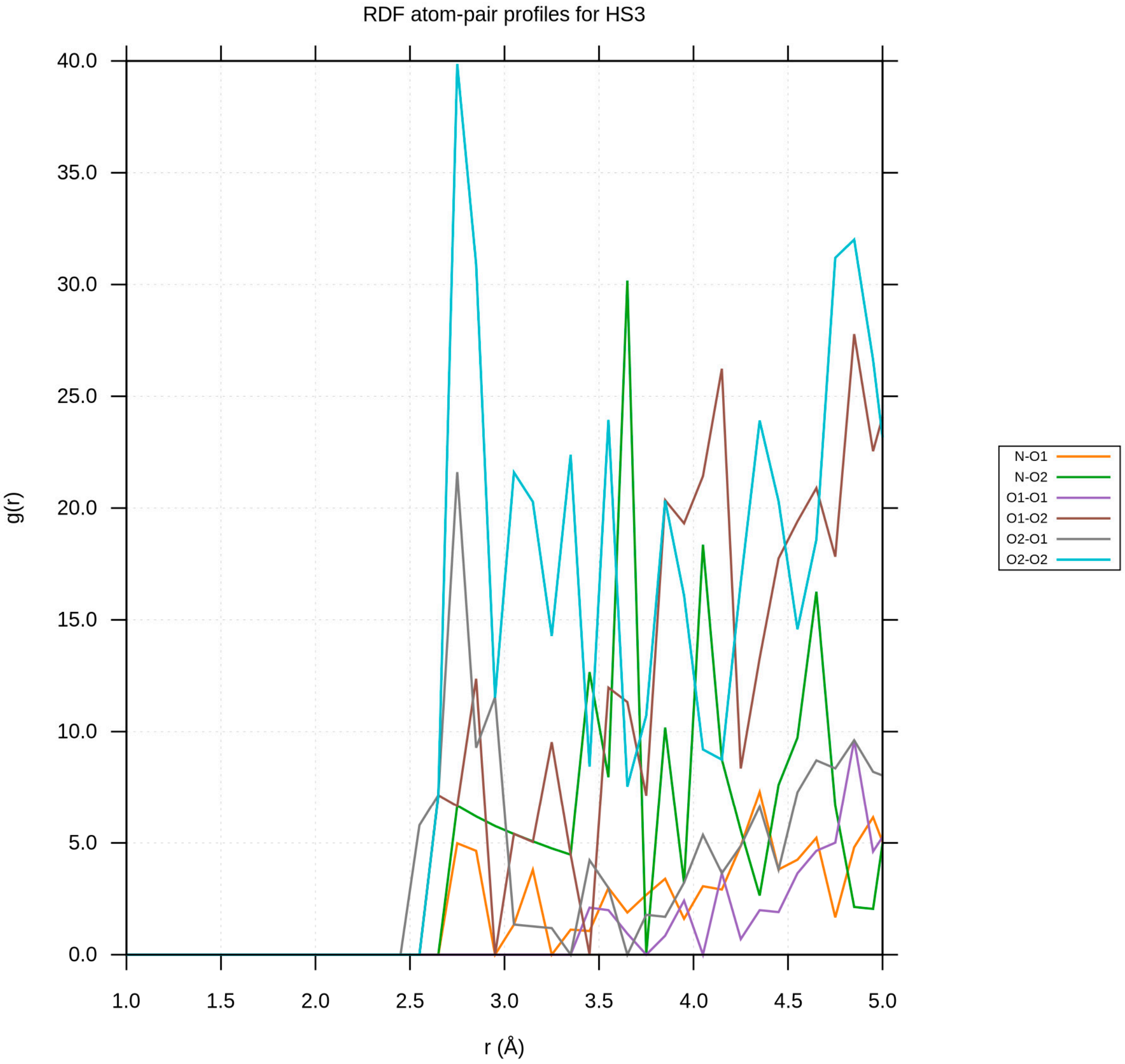

Figure S5. Atom-pair RDF profiles g(r) for IAA interactions with humic fragment HS3. Curves correspond to the available atom-type combinations involving N, O1, and O2 atoms. The displayed region (1-5 Å) covers the short-range contact domain relevant to hydrogen bonding and polar interactions. This fragment is among the three most active contributors to IAA binding.

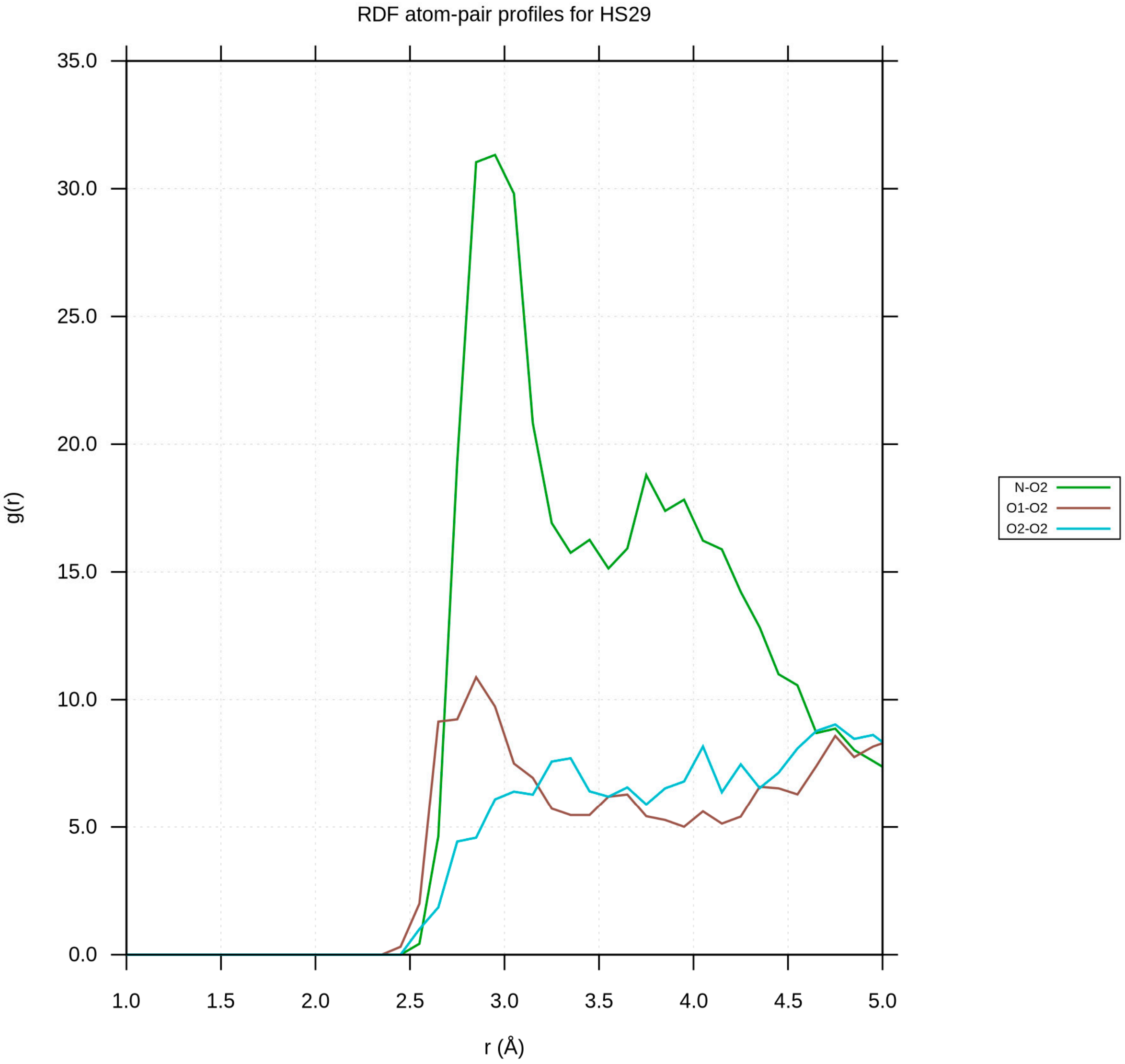

Figure S6. Atom-pair RDF profiles g(r) for IAA interactions with humic fragment HS29. Curves correspond to the available atom-type combinations involving N, O1, and O2 atoms. The displayed region (1-5 Å) covers the short-range contact domain relevant to hydrogen bonding and polar interactions.

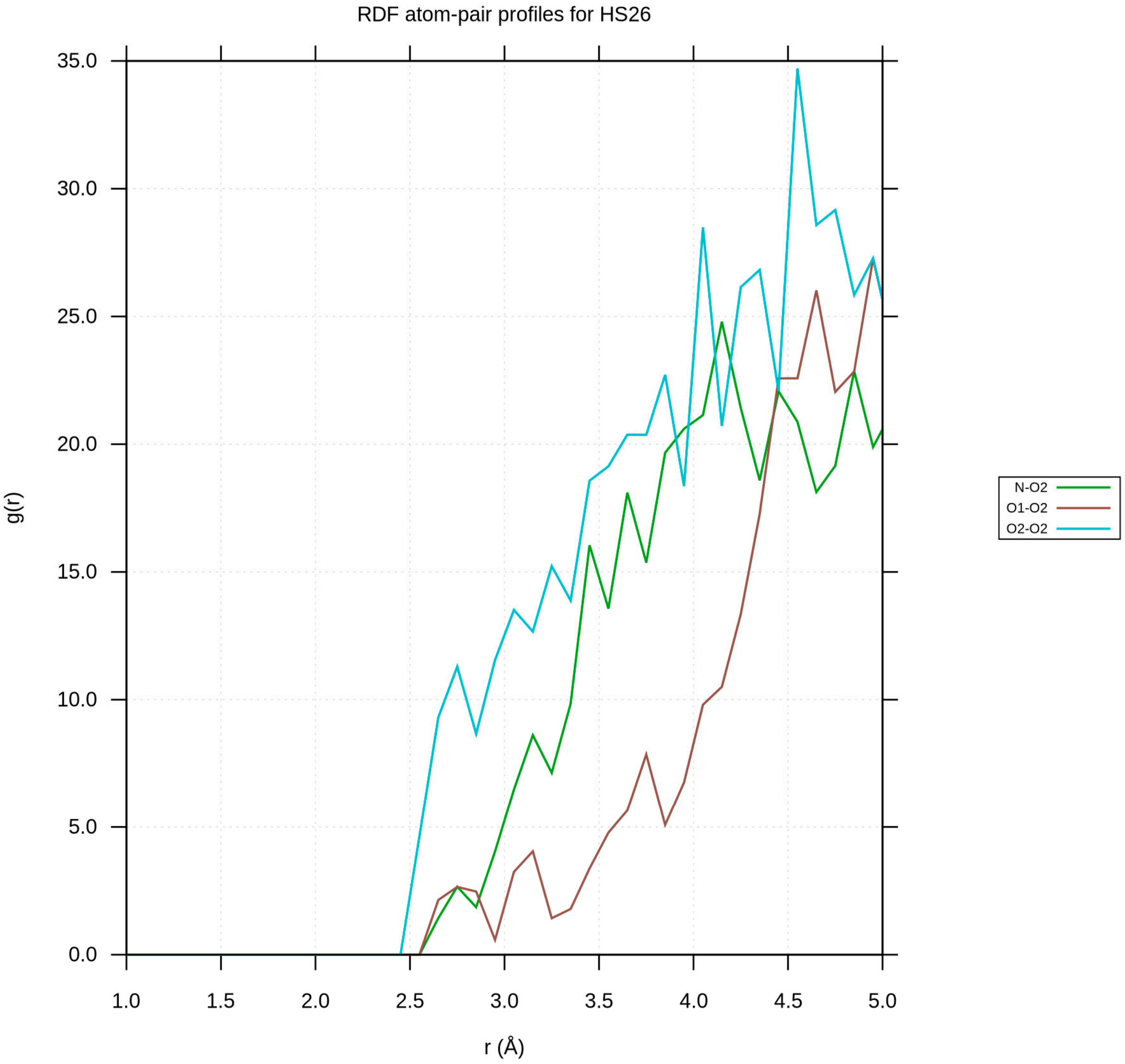

Figure S7. Atom-pair RDF profiles g(r) for IAA interactions with humic fragment HS26. Curves correspond to the available atom-type combinations involving N, O1, and O2 atoms. The displayed region (1-5 Å) covers the short-range contact domain relevant to hydrogen bonding and polar interactions.

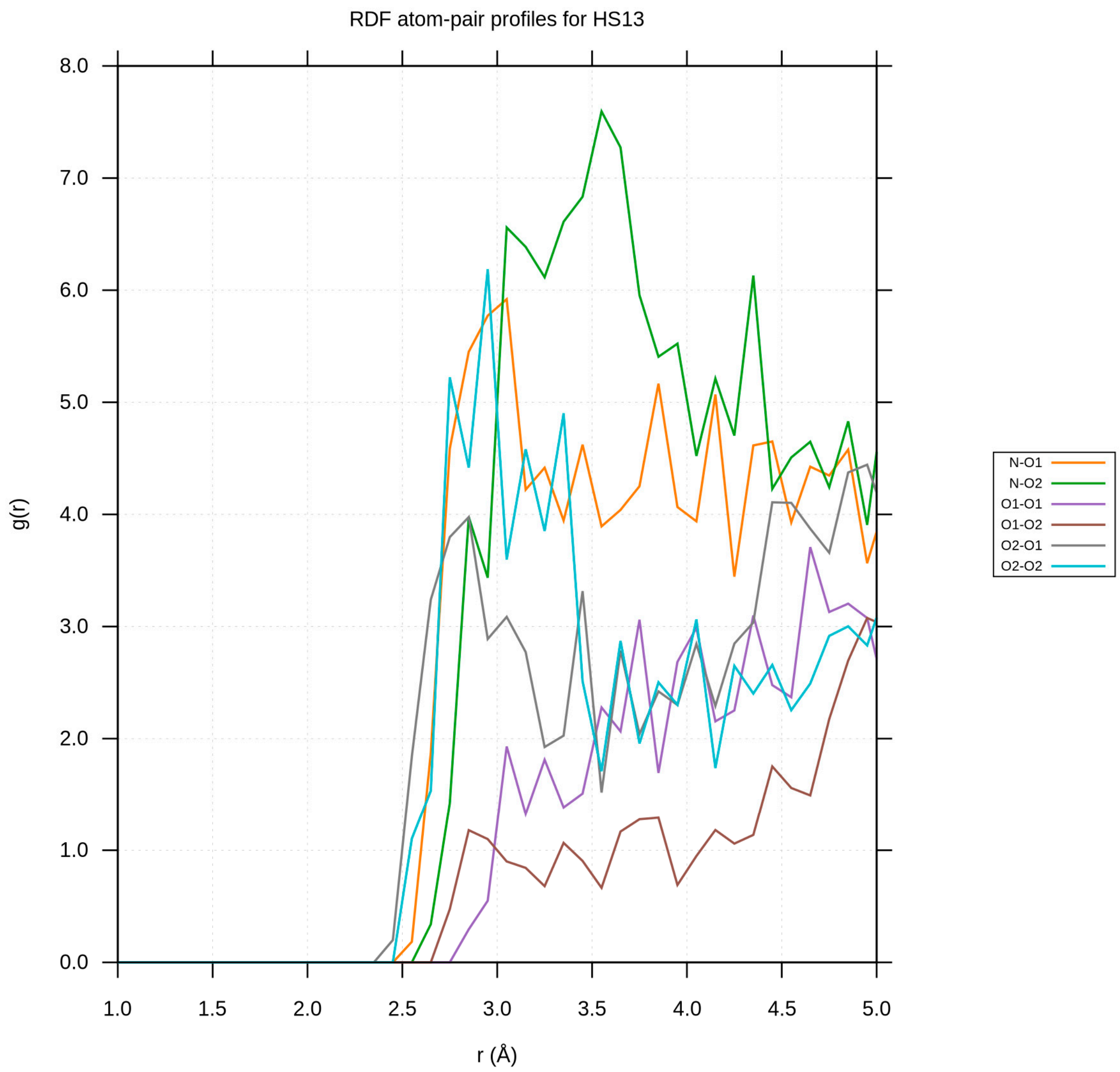

Figure S8. Atom-pair RDF profiles g(r) for IAA interactions with humic fragment HS13. Curves correspond to the available atom-type combinations involving N, O1, and O2 atoms. The displayed region (1-5 Å) covers the short-range contact domain relevant to hydrogen bonding and polar interactions.

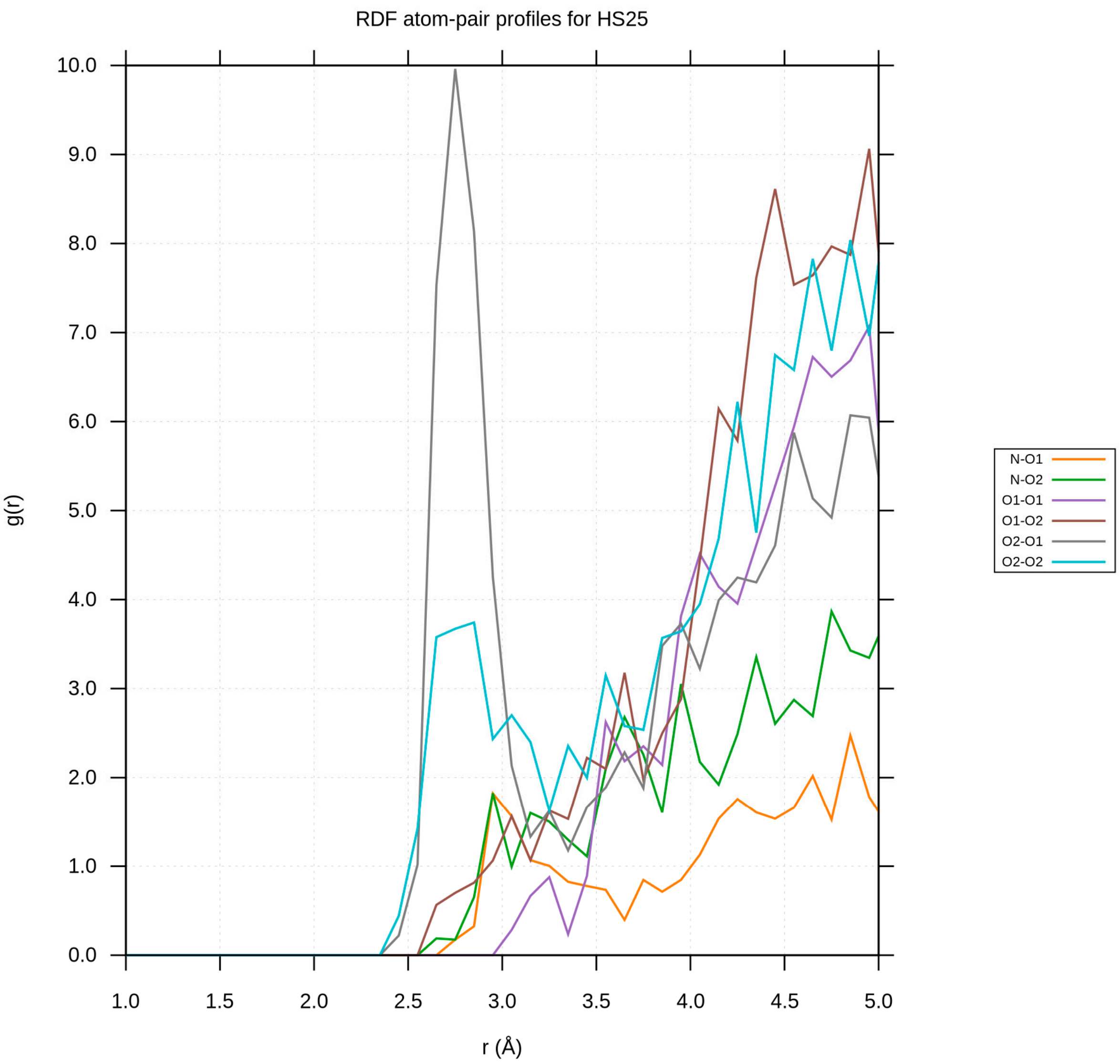

Figure S9. Atom-pair RDF profiles g(r) for IAA interactions with humic fragment HS25. Curves correspond to the available atom-type combinations involving N, O1, and O2 atoms. The displayed region (1-5 Å) covers the short-range contact domain relevant to hydrogen bonding and polar interactions.

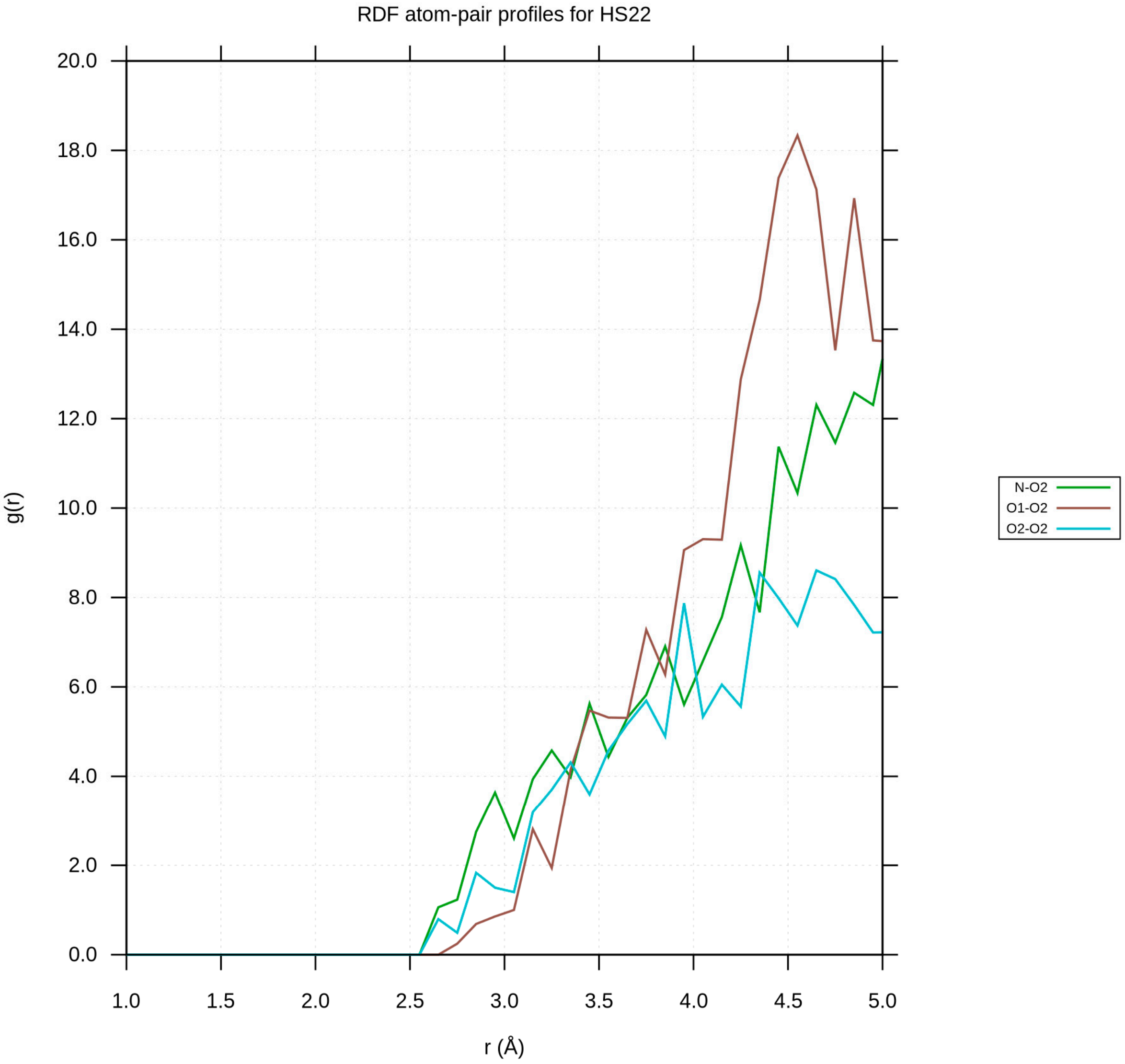

Figure S10. Atom-pair RDF profiles g(r) for IAA interactions with humic fragment HS22. Curves correspond to the available atom-type combinations involving N, O1, and O2 atoms. The displayed region (1-5 Å) covers the short-range contact domain relevant to hydrogen bonding and polar interactions.

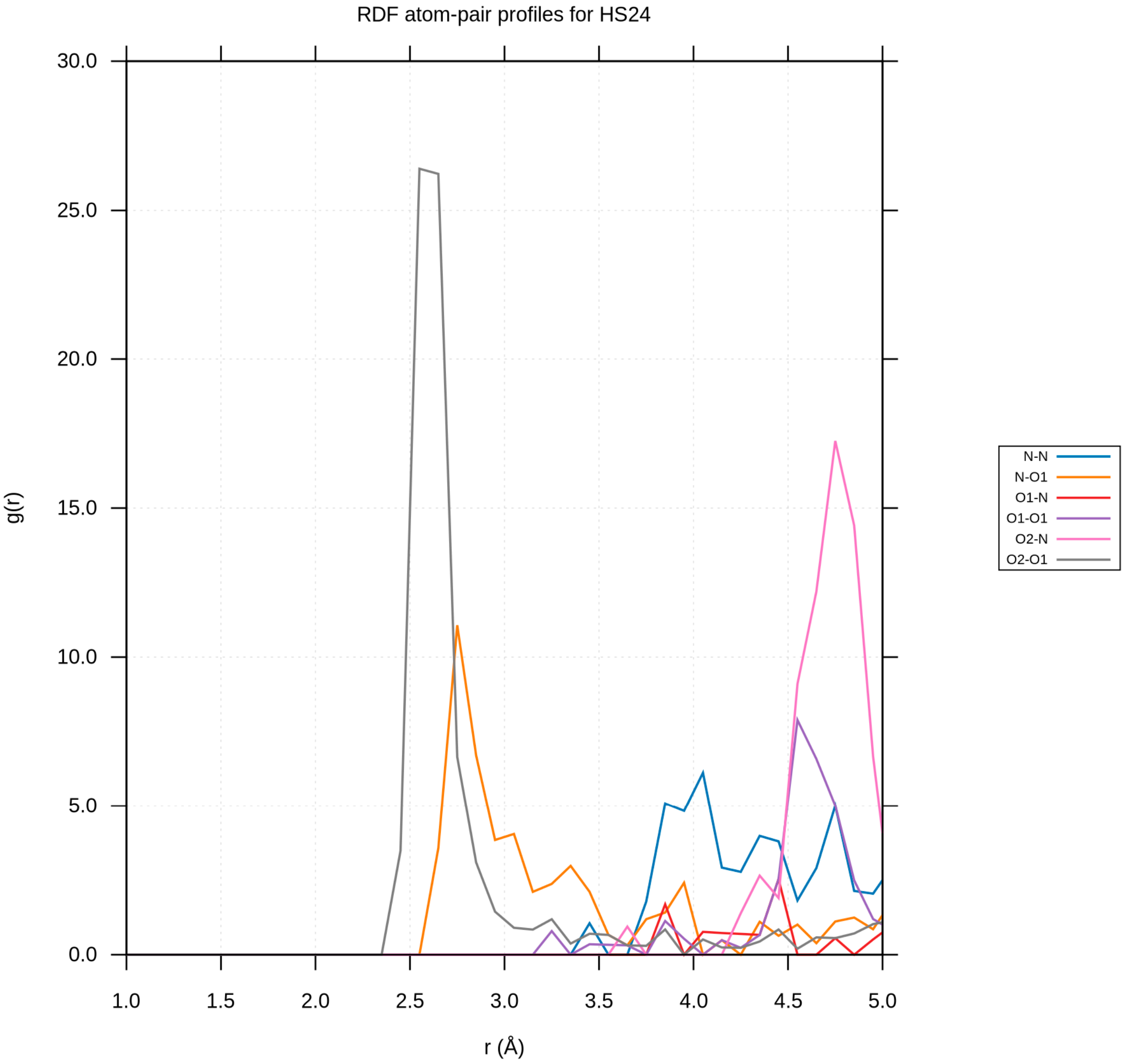

Figure S11. Atom-pair RDF profiles g(r) for IAA interactions with humic fragment HS24. Curves correspond to the available atom-type combinations involving N, O1, and O2 atoms. The displayed region (1-5 Å) covers the short-range contact domain relevant to hydrogen bonding and polar interactions.

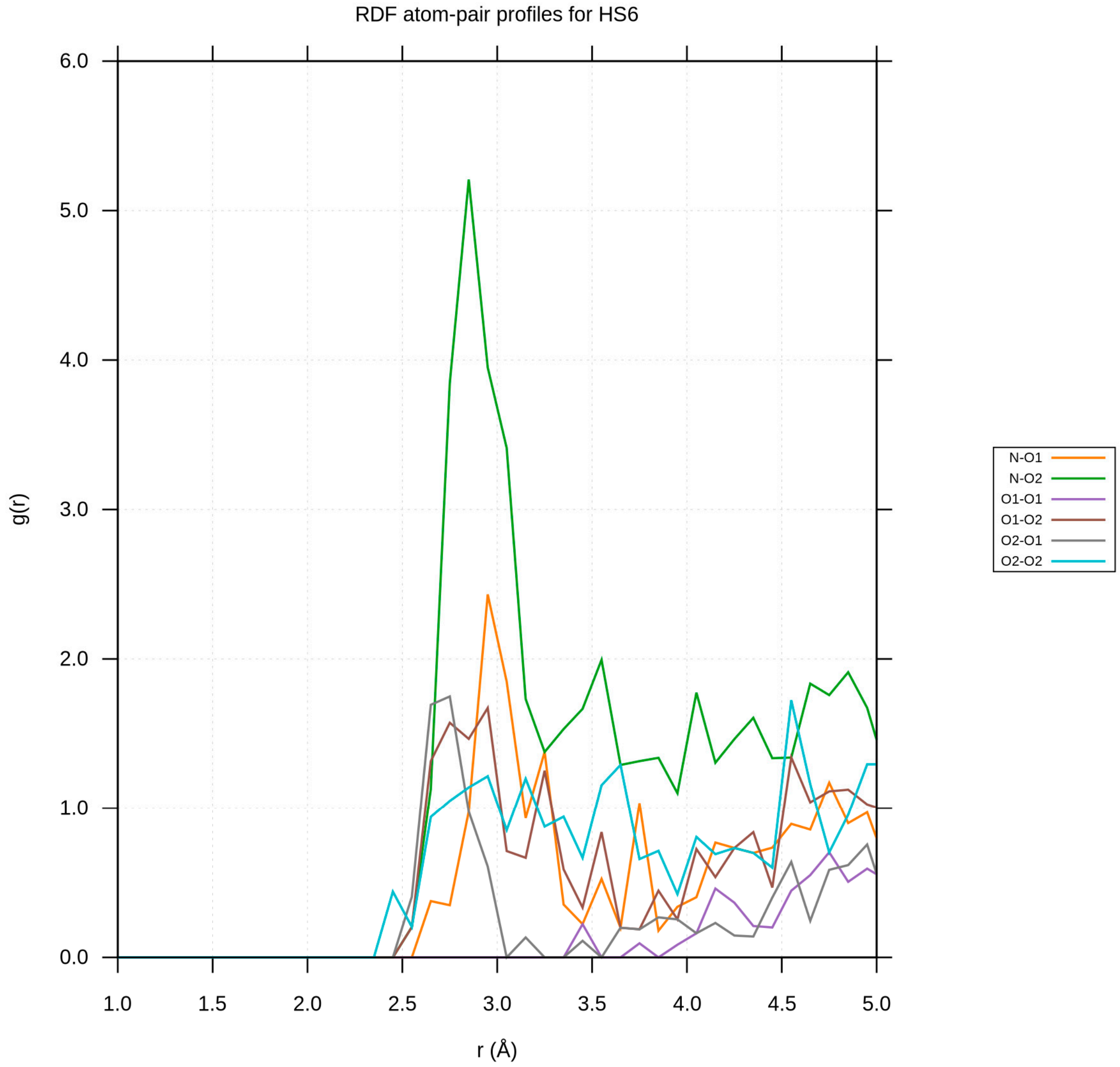

Figure S12. Atom-pair RDF profiles g(r) for IAA interactions with humic fragment HS6. Curves correspond to the available atom-type combinations involving N, O1, and O2 atoms. The displayed region (1-5 Å) covers the short-range contact domain relevant to hydrogen bonding and polar interactions.

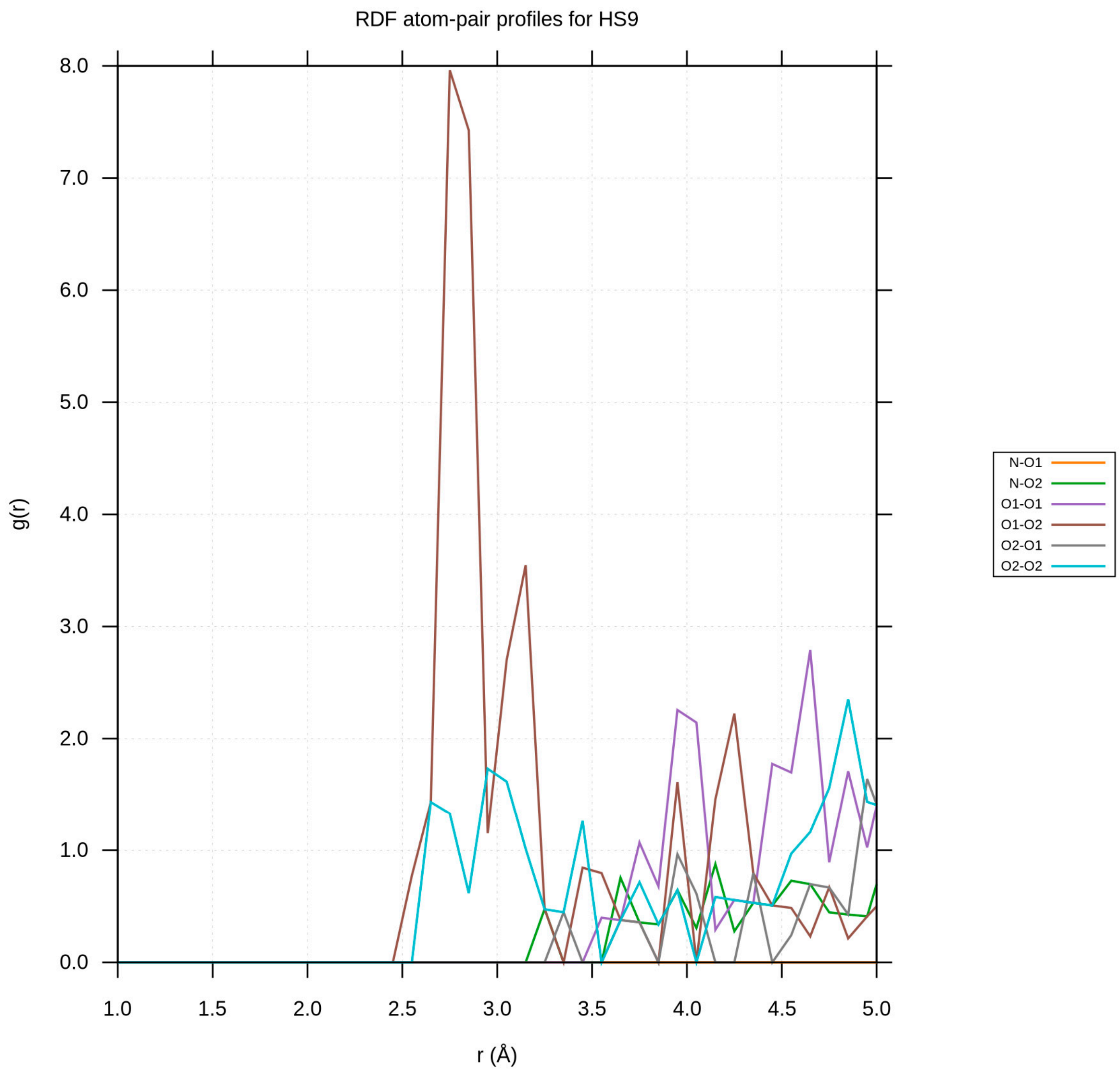

Figure S13. Atom-pair RDF profiles g(r) for IAA interactions with humic fragment HS9. Curves correspond to the available atom-type combinations involving N, O1, and O2 atoms. The displayed region (1-5 Å) covers the short-range contact domain relevant to hydrogen bonding and polar interactions.

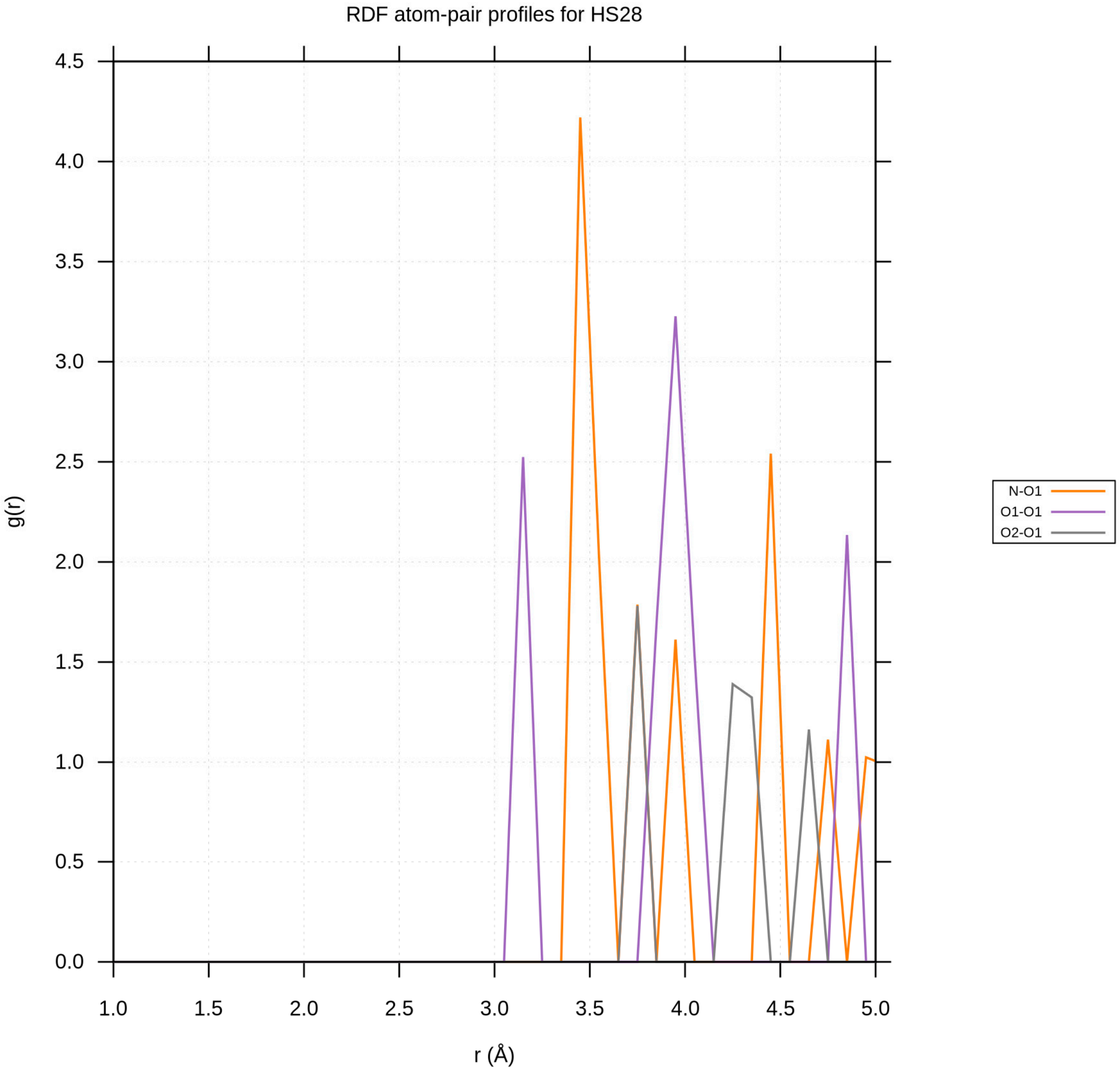

Figure S14. Atom-pair RDF profiles g(r) for IAA interactions with humic fragment HS28. Curves correspond to the available atom-type combinations involving N, O1, and O2 atoms. The displayed region (1-5 Å) covers the short-range contact domain relevant to hydrogen bonding and polar interactions.

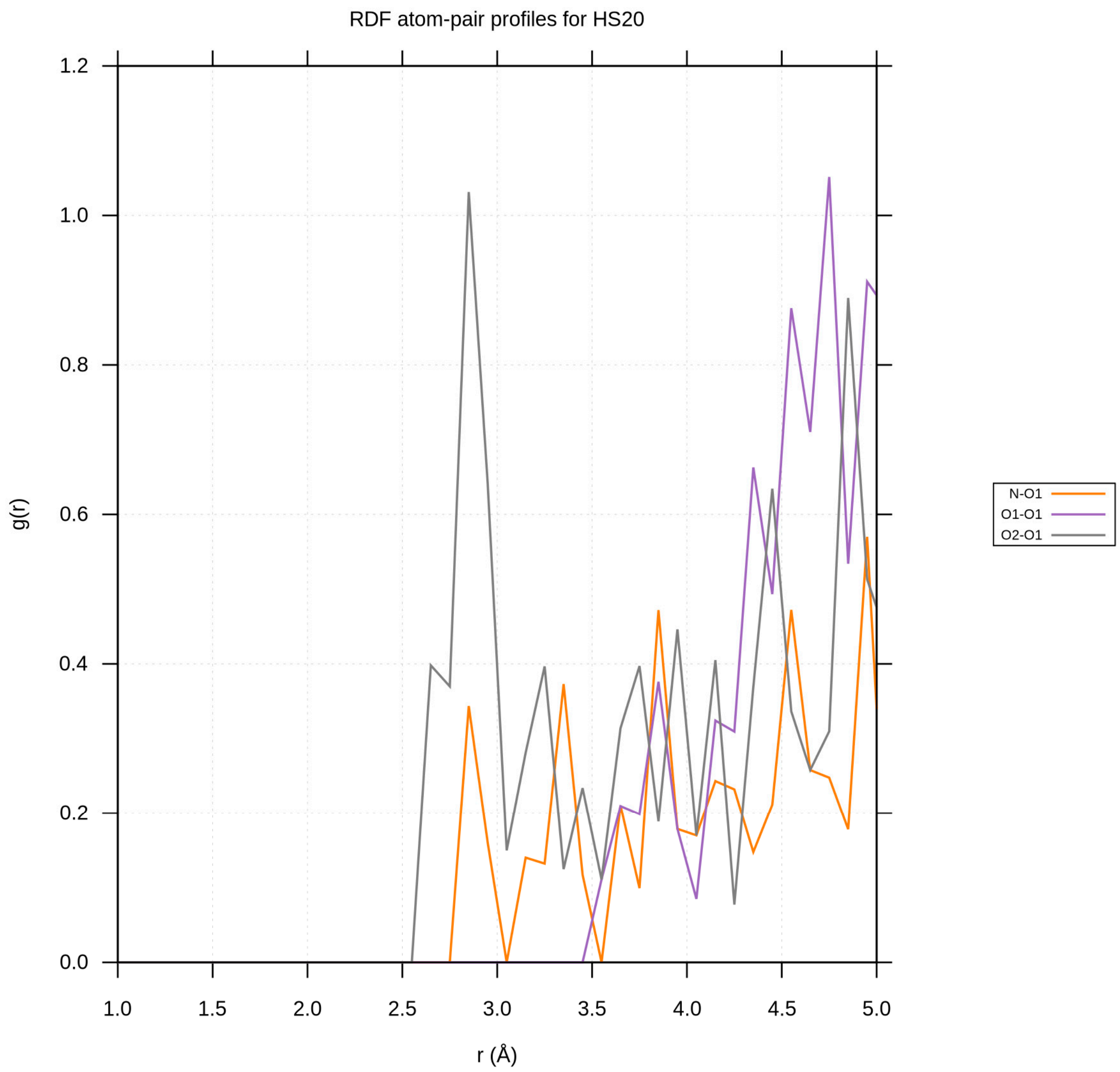

Figure S15. Atom-pair RDF profiles g(r) for IAA interactions with humic fragment HS20. Curves correspond to the available atom-type combinations involving N, O1, and O2 atoms. The displayed region (1-5 Å) covers the short-range contact domain relevant to hydrogen bonding and polar interactions.

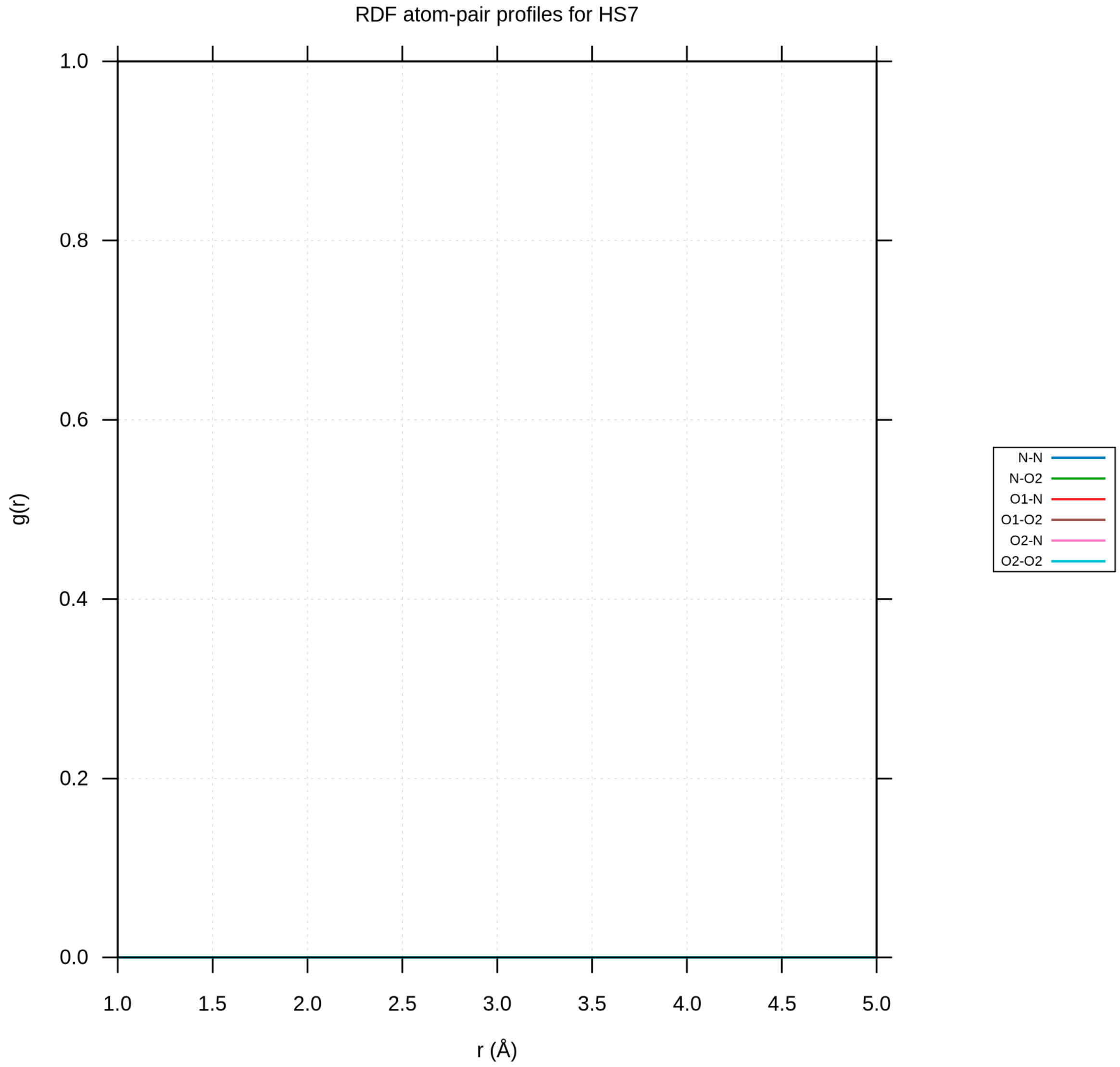

Figure S16. Atom-pair RDF profiles g(r) for IAA interactions with humic fragment HS7. No short-range atom-pair contacts satisfying the 4 Å cutoff were detected for this fragment during the analyzed trajectory; therefore, no visible RDF contact peaks are present in the 1–5 Å range. The panel is retained to document the absence of detectable IAA–HS7 contacts under the applied criterion.

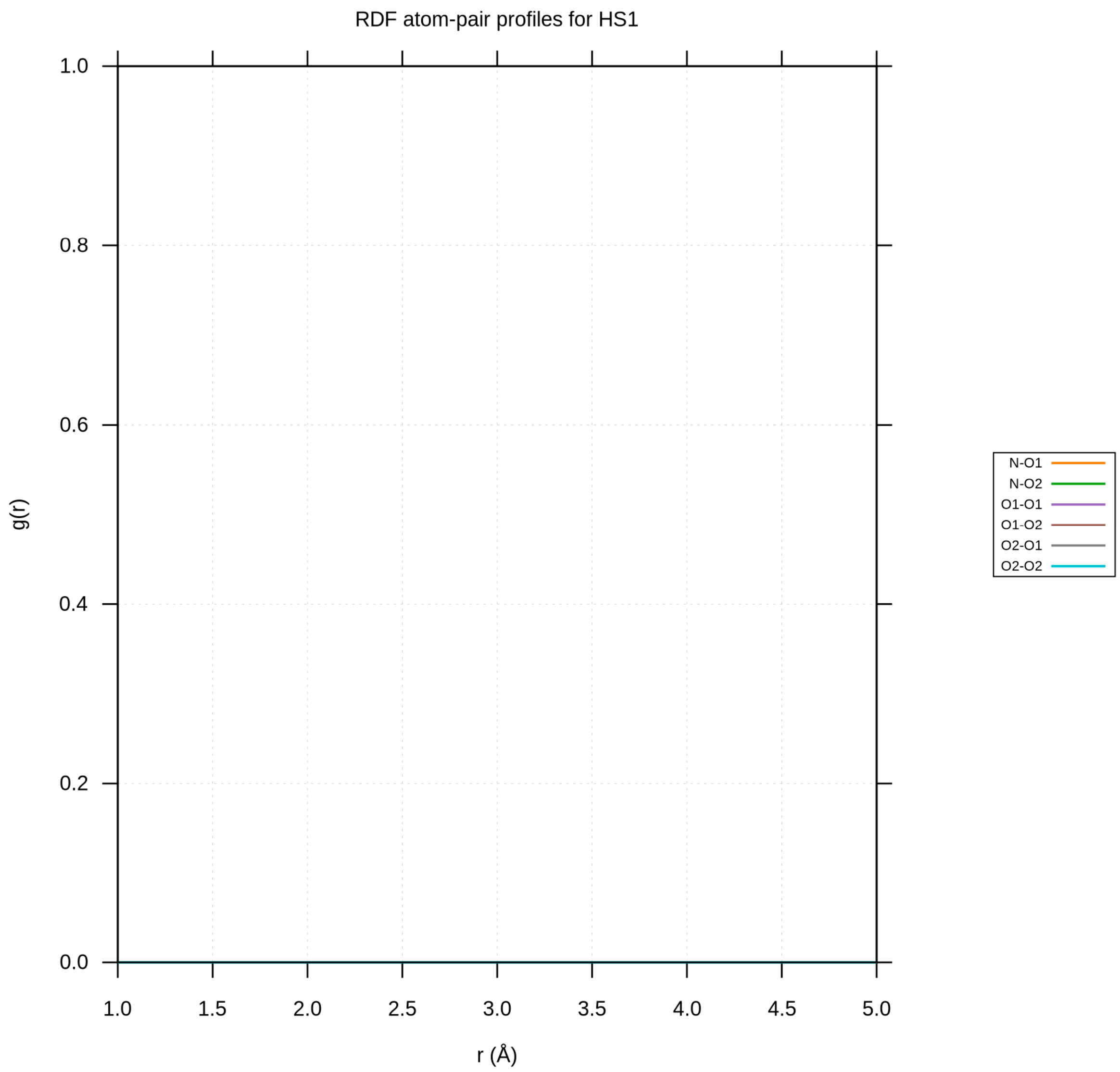

Figure S17. Atom-pair RDF profiles g(r) for IAA interactions with humic fragment HS1. No short-range atom-pair contacts satisfying the 4 Å cutoff were detected for this fragment during the analyzed trajectory; therefore, no visible RDF contact peaks are present in the 1–5 Å range. The panel is retained to document the absence of detectable IAA–HS1 contacts under the applied criterion.

S5. Interpretation of the supplementary plots

The contact-intensity matrix and RDF profiles support the conclusion that IAA binding to the humic substrate is strongly heterogeneous and localized mainly on a limited number of humic fragments. HS11 shows the largest total contact strength and broad contributions across N-O and O-O atom-pair channels. HS18 combines N- and O-mediated interactions, whereas HS3 is dominated by O-O and N-O contacts. These findings indicate that oxygen-containing functional groups provide the principal local environment responsible for IAA retention.

The r_peak map and RDF curves further show that the dominant short-range maxima are concentrated in the 2.5-3.2 Å interval, which is consistent with hydrogen bonding or strong polar contacts. Less intense contributions in the 3.2-4.0 Å range likely represent weaker polar and van der Waals contacts. Together, the supplementary heatmaps and RDF plots provide quantitative support for the molecular mechanism proposed in the main text.
